# Comprehensive characterization and comparison of aroma profiles of rambutan seed oils using GC-MS and GC-IMS combined with chemometrics

**DOI:** 10.3389/fnut.2024.1486368

**Published:** 2024-10-28

**Authors:** Yanchi Zhou, Jingtao Cui, Qiaozhu Wei, Long Wu, Tian Li, Weimin Zhang

**Affiliations:** School of Food Science and Engineering, Hainan University, Haikou, China

**Keywords:** rambutan seed oil, volatile compounds, GC-MS, GC-IMS, aroma profiles, chemometrics

## Abstract

**Background:**

Aroma significantly influences the quality of rambutan seed oil. Nonetheless, few studies have examined the volatile composition of rambutan seed oil. Thus, there is a need for a comprehensive characterization of the aroma profile of rambutan seed oil.

**Methods:**

This study systematically investigated and compared the aroma characteristics of three types (BR-4, BR-5 and BR-7) of rambutan seed oils using HS-SPME-GC-MS and HS-GC-IMS techniques, augmented by chemometric modeling.

**Results:**

GC-MS identified 135 volatile compounds, primarily hydrocarbons and esters, while GC-IMS characterized 35 compounds, mainly aldehydes and alcohols. Multivariate statistical analyses revealed significant differences in the aroma profiles among the oil samples. BR-5 exhibited the highest levels of aldehydes and hydrocarbons, whereas BR-7 demonstrated the highest content of esters and ketones. 21 and 15 differentially volatile compounds were extracted from the datasets obtained via GC-MS and GC-IMS. These compounds primarily comprised esters (floral and fruity) and aldehydes (green and fatty), interacting to create the distinctive aroma profiles of the three rambutan seed oils.

**Conclusion:**

This study provides theoretical support for evaluating the flavor characteristics and utilization potential of rambutan seed oil.

## 1 Introduction

The rambutan (*Nephelium lappaceum* L.), a tropical fruit belonging to the Sapindaceae family, originated in Malaysia, and is widely cultivated in Southeast Asian countries. It is renowned as the “king of fruits” (1). Owing to its exotic characteristics and high nutritional value, the fruit has gained popularity among consumers worldwide. Since its introduction to China in the 1960s, Baoting Li and Miao Autonomous County (Hainan, China) has emerged as the largest rambutan producer in the country, boasting a plantation area of up to 39,000 acres, owing to its climatic resemblance to Malaysia and Thailand (2). Rambutan “BR” series is a series of varieties that can be popularized and cultivated by Baoting Tropical Crops Research Institute after decades of cultivation and development. In the Chinese market, BR-4, BR-5, and BR-7 are the top three popular rambutan varieties, prized for their refreshing flavor and pleasant aroma. Typically, rambutan is consumed fresh. Additionally, canned rambutan juice, jam, and syrup are the primary industrial products derived from rambutan. After direct consumption or industrial processing, the residues are mainly seed (9.5% of whole fruit) and peel (45.7%) discarded as by-products (1). Recently, researchers reported that, on average, 1,900 tons of rambutan seeds are discarded annually as by-products in Thailand (3). These industrial by-products are generated in large quantities and, if not effectively utilized, could result in significant environmental problems and economic losses.

Rambutan seeds show promise as a novel nutrient source for human diets and food applications due to their high levels of crude fat (33.4–39.13%), protein (7.8–12.4%), carbohydrates (46–48.10%), dietary fiber (11.6%), and ash (1.22%) (1). Additionally, rambutan seeds are of significant interest due to their bioactive compounds, including antioxidants and phenolic compounds (4). Previous studies have documented elevated levels of oleic acid (42.00%) and arachidonic acid (34.30%) in rambutan seed oil (5). Palanisamy et al. (6) examined the composition of phytosterols and tocopherols in rambutan seed oil. Furthermore, Solís-Fuentes et al. (7) investigated the thermodynamic behavior of rambutan seed oil, showcasing its potential as a substitute for confectionery fats and cocoa butter due to its distinctive fatty acid profile. Nonetheless, few studies have examined the volatile composition of rambutan seed oil (8, 9). Aroma plays a key role in shaping the overall acceptability of edible oil and may be affected by variety differences. Thus, there is a need for a comprehensive characterization of the aroma profile of rambutan seed oil.

Numerous researchers have identified certain aroma compounds, including esters, aldehydes, alcohols, and furans, as contributors to the unique aroma of edible oils (10, 11). Likewise, aroma can serve as a pivotal indicator for distinguishing between various varieties of vegetable oils (12). Currently, headspace-solid phase microextraction-gas chromatography–mass spectrometry (HS-SPME-GC-MS) and headspace-gas chromatography-ion mobility spectrometry (HS-GC-IMS) techniques are the most commonly employed methods for analyzing volatile compounds in food matrices due to their features of requiring no sample pre-treatment, high sensitivity, and rapidity (10, 13). These techniques have been extensively utilized in the analysis of food composition (14), detection of food adulteration and traceability (15, 16), and assessment and enhancement of food quality (13). When GC-MS and GC-IMS are combined, they can make up for their shortcomings and give full play to their respective advantages. This combined technique not only expands the detection range of volatile components in the sample, but also improves the accuracy and reliability of the analysis. To the best of our knowledge, no studies have integrated GC-MS and GC-IMS techniques to characterize the volatile compound profiles of rambutan seed oils comprehensively.

Herein, a comprehensive analysis and comparison of the volatile compound profiles of the extracted oils from the three most common varieties of rambutan in Chinese (namely, BR-4, BR-5, and BR-7) was carried out using a combination of GC-MS and GC-IMS techniques. Chemometric modeling was employed to deepen the understanding of the differences in aroma characteristics among the three rambutan seed oils and to identify biologically significant differential aroma compounds. This study aims to establish a theoretical and scientific foundation for the comprehensive development and utilization of rambutan seed oil in the realm of deep processing.

## 2 Methods

### 2.1 Materials, chemicals, and reagents

The three rambutan varieties (BR-4, BR-5, and BR-7) of the “BR” series were sourced from the Baoting Rambutan Germplasm Resource Park in Hainan Province, China, where all rambutan varieties are cultivated and supervised according to standardized practices. Following harvest, rambutan fruits were promptly transported to the laboratory for processing, involving the separation of pulp, peel, and seeds. The seeds were dried in a constant temperature blast oven (DHG-9013A, Shanghai Yiheng Scientific Instrument Co., Ltd., Shanghai, China) at 50°C for 24 h, ground using a grinder (FW80, Cangzhou Tuoyan Experimental Instrument Co., Ltd., Cangzhou, China), and subsequently sieved through a 50-mesh sieve. Subsequently, the powdered samples were vacuum-sealed and stored in a refrigerator at −20°C until further analysis.

Methanol (chromatographic purity), isopropanol (chromatographic purity), acetonitrile (chromatographic purity) and 2,4,6-trimethylpyridine (GC grade) as an internal standard for GC-MS were procured from ANPEL Laboratory Technologies (Shanghai) Inc. (Shanghai, China). Methyl tert-butyl ether (chromatographic purity) and acetic acid (chromatographic purity) were acquired from Shanghai Aladdin Biochemical Technology Co., Ltd. (Shanghai, China).

### 2.2 Samples of rambutan seed oils

The rambutan (*Nephelium lappaceum* L.) seed oil extraction followed the method described by Matyash et al. (17) with slight adaptations. Specifically, 500 mg of powdered sample was precisely weighed and transferred into a 50 mL centrifuge tube. Subsequently, 4 mL of distilled water and 9.6 mL of methanol- methyl tert-butyl ether solution (1:5, v/v) were added. After vortexing for 1 min, the mixture underwent sonication in an ice-water bath for 5 min, repeated thrice. The mixture was then cooled to −40°C and left to stand for 60 min before centrifugation at 3000 rpm for 15 min. Finally, the supernatant was collected, evaporated under nitrogen to obtain oils, sealed, and stored at −20°C for further analysis.

### 2.3 HS-SPME-GC-MS analysis

The volatile components of rambutan seed oil samples were analyzed by HS-SPME-GC-MS. Briefly, a 3 g oil sample was introduced into a 20 mL headspace bottle fitted with a cap featuring a silicone/polytetrafluoroethylene septum, with a DVB/CAR/PDMS 50/30 μm 2 cm fiber inserted. Oil samples were equilibrated for 5 min and then extracted at 50°C for 50 min with shaking at 250 rpm.

Separation and identification of volatile compounds were performed using an Agilent 6890A/5973C gas chromatography–mass spectrometry system equipped with a DB-WAX capillary column (30 m × 0.25 mm, film thickness 0.25 μm, Agilent, United States). The oven temperature program comprised an initial hold at 40°C for 1 min, followed by an increase to 120°C at a rate of 3°C/min for 5 min, then to 250°C at a rate of 5°C/min, and finally maintained at 250°C for 5 min. The inlet temperature and flow rate were set at 250°C and 1 mL/min, respectively. Mass spectrometry parameters were configured as follows: electron ionization mode, ion source temperature of 250°C, ionization energy of 70 eV, transmission line temperature of 240°C, and ion mass scan range of 40–600 m/z.

Retention indices (RIs) were determined using n-alkanes (C5–C23) as an external reference. Volatile compounds were identified by comparing mass spectral information and RIs with those from the NIST 20.L database (National Institute of Standards and Technology, Gaithersburg, MD, United States). The chemical structure most closely matching the mass spectrometry information and RI value was chosen as the best identification. Peak intensities of volatile compounds are essential for elucidating aroma differences among various rambutan seed oils. The concentration of volatile components (C_i_) were calculated according to the following formula:


$$C_{i}=\left(C_{\mathrm{is}}\times A_{i}\right)/A_{\mathrm{is}}$$

Where C_is_, A_i_ and A_is_ were the concentration of internal standard (IS), peak area of determined components, and peak area of IS.

### 2.4 HS-GC-IMS analysis

GC-IMS-based analysis of volatile compounds was performed using an Agilent 8,090 gas chromatography system equipped with a G.A.S. ion mobility spectrometer and a PALRSL85 headspace automatic sampler. The instrument was equipped with an HP-5 capillary column (30 m × 0.32 mm, film thickness 0.25 μm, Agilent, United States). Three grams of oil samples were precisely weighed into a 20 mL headspace bottle and incubated at a constant temperature of 60°C for 15 min with agitation at 500 rpm. Subsequently, a headspace needle (80°C) aspirated 300 μL of headspace gas for analysis. Compounds were separated using an HP-5 capillary column (30 m × 0.32 mm, film thickness 0.25 μm, Agilent, United States) with a column temperature maintained at 60°C. The ion mobility spectra temperature was set to 45°C. High-purity nitrogen served as the carrier gas with a flow rate starting at 2 mL/min for 2 min, increasing to 10 mL/min over 8 min, then to 100 mL/min over 10 min, and finally to 150 mL/min over 10 min. The drift gas flow rate was maintained at 150 mL/min. Identification of volatile compounds was achieved by comparing the RIs and drift times with standards from the GC-IMS retrieval library (G.A.S., Dortmund, Germany). Quantitative results were expressed as relative abundance (10). The aroma profiles of seed oils from three rambutan varieties were analyzed using the analytical software (VOCal) and accompanying plug-ins (Reporter, GalleryPlot) to explore various perspectives. Spectral differences between samples (2D top view, 3D spectra, and difference spectra) were directly compared using the Reporter plug-in. The Gallery Plot plug-in was used to visually compare fingerprints and observe differences in VOCs between samples.

### 2.5 Statistical analysis

Each trial was conducted in triplicate, and results were expressed as mean ± standard deviation. Analysis of variance (ANOVA) was employed to determine differences in aroma compound content among the analyzed varieties, followed by the Tukey test as a post-hoc analysis. Significance was established at a probability of 0.05 (*p* < 0.05) using the least significant differences procedure. ANOVA was conducted using SPSS 26.0 statistical software (SPSS Inc., Chicago, IL, United States). Multivariate statistical analysis, including principal component analysis (PCA), orthogonal partial least squares discriminant analysis (OPLS-DA), and hierarchical cluster analysis (HCA), was performed using Metaboanalyst 5.0 software and SIMCA 14.1 (Umetrics, Umea, Sweden). Additional data analyses and image plotting were performed using Excel (Microsoft, United States) and OriginPro 2021 (OriginLab Corporation, Northampton, Massachusetts, United States).

## 3 Results and discussion

### 3.1 Overview and difference of volatile compounds identified by HS-SPME-GC-MS in rambutan seed oils

**Figure 1:**
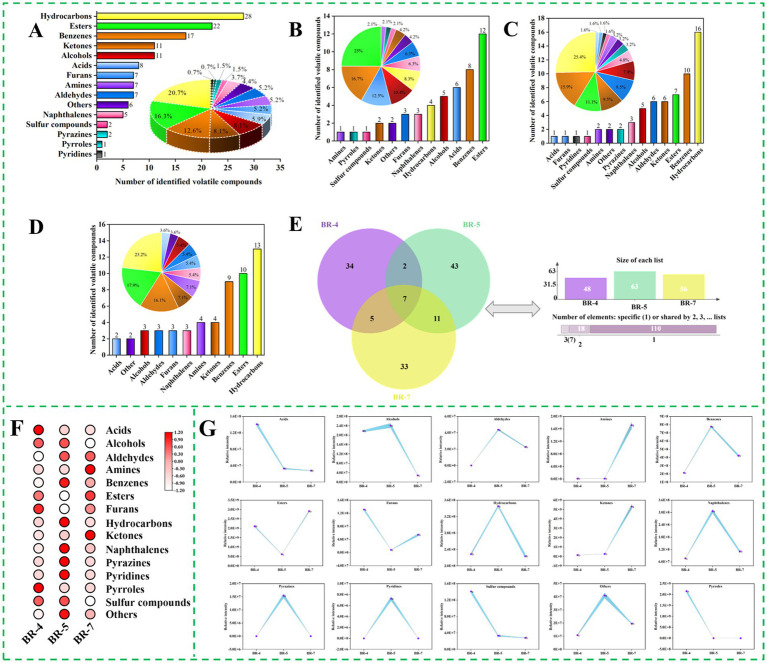
Overview of volatile compounds identified by GC-MS in rambutan seed oils. Total numbers of volatile compounds identified in rambutan seed oils **(A)**. Numbers and percentages of volatile compounds in BR-4 **(B)**, BR-5 **(C)**, and BR-7 **(D)**. Venn plot of volatile compounds in three rambutan seed oils **(E)**. Heat map of different volatile compound species 111 rambutan seed oils **(F)**. Intensity of different species of volatile compound 111 rambutan seed oils **(G)**. The different letters in the bar chart were shown as a significant difference between samples (*p* < 0.05).

The volatile component profile significantly influences the aroma and organoleptic quality of foods, particularly edible oils, thereby influencing consumer preference. Rambutan seed oil samples were analyzed for volatile compounds using HS-SPME-GC-MS. A total of 135 volatile compounds were identified in rambutan seed oils, including 28 hydrocarbons, 22 esters, 17 benzenes, 11 alcohols, 11 ketones, 8 acids, 7 aldehydes, 7 amines, 7 furans, 6 others, 5 naphthalenes, 2 pyrazines, 2 sulfur compounds, 1 pyridine, and 1 pyrrole (Figure 1A; Supplementary Table S1). The major volatile compounds included hydrocarbons (20.74%), esters (16.30%), benzenes (12.59%), and alcohols (8.15%) (Figure 1A). Similarly, Khairy et al. (8) identified alcohols (23.15%), hydrocarbons (17.65%), and esters (16.49%) as significant volatile compounds in Malaysian rambutan seed oil. However, large amounts of pyrazine (29.38%) and acids (23.14%) were also observed in their study, which is different from our results. These discrepancies could be attributed to variations in rambutan variety, origin, and growing conditions.

The quantities of volatile compounds identified varied significantly among the three rambutan seed oils, as depicted in Figures 1B–D. BR-5 exhibited the highest number of volatiles (63 varieties), whereas BR-4 had the lowest (48 varieties). Additionally, the composition of aroma compounds differed among the samples. This indicates that the three rambutan seed oils analyzed in this study possessed distinct aroma profiles. The shared volatile compounds among the three rambutan seed oils were represented in a Venn diagram (Figure 1E), revealing a total of 7 compounds, including p-xylene, 1-ethyl-3,5-dimethylbenzene, 1-ethyl-2,4-dimethylbenzene, 1,2,3,5-tetramethylbenzene, 4-fluoro-2-nitrophenyl methyl phthaloate, sec-butyl acetate, and naphthalene. There were 34, 43, and 33 volatile compounds observed exclusively in BR-4, BR-5, and BR-7, respectively. 2,3-dimethylpentane and 1,2,3,4-tetrahydro-6-methylnaphthalene were detected in both BR-4 and BR-5. The 2-methylheptanoic acid, ethanol, benzene, dodecanoic acid, methyl ester, and oxalic acid, butyl cyclobutyl ester were present in both BR-4 and BR-7. Variations in rambutan varieties and environmental conditions may account for the presence of different volatile compounds in the three rambutan seed oils.

To explore variations in aroma substance content among different rambutan seed oils, each aroma molecule was categorized, and the peak area of all molecules within each category was summed to represent their relative contents, as shown in Figures 1F,G. Esters exhibit diverse odor and flavor properties and are prevalent as major constituents of fruits and essential oils, imparting fruity, sweet, floral, and honey sensations to food. They are synthesized through the reaction of alcohols or phenols with acids or their derivatives (18). BR-7 exhibited significantly higher levels of ester compounds compared to BR-4 and BR-5 (Figure 1G). L-proline, 5-oxo-, n-propyl ester, and propanoic acid, ethenyl ester were the predominant esters in BR-4, absent in BR-5 and BR-7 (Supplementary Table S1). Sec-butyl acetate and pentyl 3-methylbutanoate were the most abundant esters in BR-5 and BR-7, respectively. Sec-butyl acetate was reported in *Solanum lycopersicum* L (19) and *Zingiber officinale* (20), while Pentyl 3-methylbutanoate was found in *Humulus lupulus* (21) and *Capsicum annuum* (22), both commonly used in flavoring. The content of other ester molecules varied among the three rambutan seed oils according to the variety. Similarly, a previous study documented the presence of abundant esters in fermented rambutan seeds (9).

Hydrocarbons play a crucial role as flavor components in various foods, such as sesquiterpenes commonly present in tea (23). Hydrocarbons typically result from free radical reactions involving the decomposition of fatty acids. In addition to cis-trans isomerization, isomerization of the carbon–carbon double bond (C=C) position in the intermediate product facilitates the formation of a diverse range of hydrocarbons (24). The concentration of this aroma substance was notably higher in BR-5 compared to the other samples, as shown in Figure 1G. BR-4 contained only four hydrocarbon molecules, namely azetidine, 1,2-dimethyl-, (3E,7E)-4,8,12-Trimethyltrideca-1,3,7,11-tetraene, pentane, 2,3-dimethyl-, and nonane, 2,6-dimethyl-. The most predominant hydrocarbons in BR-5 were 1,3-Hexadien-5-yne and 1H-Indene, 2,3-dihydro-5-methyl-, with the former exclusive to this group (Supplementary Table S1). The most abundant hydrocarbons in BR-7 were undecane, 3,8-dimethyl-, Undecane, 4,6-dimethyl-, and octane, 2,3,6-trimethyl-. Thanks to its distinctive odor, undecane, 3,8-dimethyl-, is utilized as an ingredient in fragrances. Apart from the effect of variety, the pre-treatment method may also affects the concentration of hydrocarbons in rambutan seed oil. Previous research on rambutan seeds (9) and cacao beans (25) demonstrated that roasting decreased the levels of hydrocarbons in the samples.

Ketones commonly originate from lipid oxidation, Maillard reactions, and amino acid degradation, profoundly influencing the aroma profile of food products. Saturated ketones typically impart fruity, cheesy, and fatty flavors, whereas diketones contribute to the sweet, buttery, and caramelly sensations in coffee (26). Our analysis revealed variations in ketone composition among the three rambutan seed oils. BR-7 exhibited the highest ketone content, whereas BR-4 had the lowest (*p* < 0.05), as illustrated in Figure 1G. The predominant ketones in BR-4, BR-5, and BR-7 were 2-Imidazolidinone, 3-Heptanone, 2-methyl-, and 2-Pyrrolidinone, 5-(cyclohexylmethyl)-, respectively.

Alcohol compounds in foods typically impart flavors and sensations like sweetness, fruitiness, mellowness, aroma, and freshness. Linear alcohols commonly result from lipid oxidation, while microbial degradation of branched-chain aldehydes primarily produces branched-chain alcohols (18). Figure 1G illustrates a notable disparity in alcohol content among the three rambutan seed oils, with BR-5 exhibiting significantly higher levels than BR-4 and BR-7. Specifically, ethanol represented the predominant alcohol compound in BR-4 and BR-7, while 2-octanol and benzeneethanol are the most important components in BR-5 (Supplementary Table S1). Similar results were reported by Khairy et al. (8), identifying ethanol, 2-(1-methylethoxy)- and 1-octanol as significant alcohols in rambutan seed oil from Malaysia. Alcohol compounds actually contribute minimally to food flavor due to their higher odor thresholds (18).

Furans originate from the oxidation of dehydrated carbohydrates and fatty acids, or through the Amadori rearrangement mechanism, primarily contributing to a caramel-like flavor (26). Additionally, these compounds can be utilized for the production of butanediol and scopolamine. Seven furans were identified in rambutan seed oils (Supplementary Table S1). BR-4 contained 3,4-diacetylfurazan, 2-ethyltetrahydrofuran, and 2-butyl-3-(4-.beta.-diethylaminoethoxybenzoyl)benzofuran as the main furan compounds, while BR-7 exhibited 5-methyl-2-(2-methyl-2-tetrahydrofuryl)tetrahydrofuran, 3-methyl-(3 h)-isobenzofuran-1-one, and 2-acetyl-2-methyltetrahydrofuran. Dibenzofuran was the sole furan identified in BR-5. Previous studies have demonstrated that derivatives of dibenzofuran possess anti-inflammatory, hemostatic, and vasodilatory properties (27). Furans may significantly contribute to the aroma of rambutan seed oils due to their low aroma threshold.

Volatile sulfur compounds typically emit unpleasant odors, such as those reminiscent of onion and putrefaction. Methanesulfonyl chloride and 1-propanamine, 3-dibenzo[b,e]thiepin-11(6 h)-ylidene-n,n-dimethyl-, s-oxide were detected in BR-4 and BR-5, respectively (Supplementary Table S1). BR-7 did not exhibit any sulfur-containing volatile compounds. Previous studies on rambutan seeds and their oils have not reported the presence of sulfur compounds (8, 28). Interestingly, previous research has identified elevated levels of sulphides in cold-pressed rapeseed oil (29) and roasted sesame oil (30). Similarly, to our study, the seed coat of rapeseed and sesame seeds was also not stripped during the pretreatment process, which may explain to some extent the presence of sulphides in the rambutan seed oil analyzed in this study.

Besides the aforementioned compounds, rambutan seed oil also contained acids, aldehydes, amines, benzenes, naphthalenes, pyrazines, pyridines, pyrroles, and other aroma constituents. These components exhibited notable variations among the three rambutan seed oils. Specifically, pyridines were exclusively present in BR-5, while pyrroles were solely detected in BR-4. Moreover, the levels of naphthalenes were significantly higher in BR-5 compared to the other two samples (Figure 1G). Overall, significant differences in both the types and relative concentrations of aroma substances were evident among the three rambutan seed oils based on GC-MS analyses.

### 3.2 Multivariate statistical analysis of volatile compounds in rambutan seed oils based on HS-SPME-GC-MS

To further compare the aroma profiles of different rambutan seed oils and to detect intervarieties differences, multivariate statistical analyses of the samples were performed (15). Initially, a PCA model was employed to discern overall differences between the oil varieties. The 3D score plot (Supplementary Figure S1A) revealed distinct clusters, indicating significant variations in aroma profiles. Components 1 and 2 explained 53.70 and 46.00% of the total variance, respectively, with an *R*^2^X_(*cum*)_ of 0.997. Loading and biplot plots (Supplementary Figures S1B,C) further elucidated variable contributions. The contribution of ethanol was higher in BR-4 than in BR-5 and BR-7. Naphthalene, 1,2,3,4-tetrahydro-6-methyl- was present in BR-5 to a greater extent than in the other oil samples. The 2-methylheptanoic acid was a contributor to facilitate the differentiation of BR-7 from BR-5 and BR-4. Subsequently, an OPLS-DA model, a supervised learning model, was run to delve deeper into volatile compound differences. The 3D score plot (Supplementary Figure S1D) echoed PCA model trends, with *R*^2^X_(*cum*)_ = 0.997, *R*^2^Y_(*cum*)_ = 1, and Q^2^ = 1, supported by robust alignment test results (*R*^2^ = 0.161, Q^2^ = −0.645) (Supplementary Figure S1E). GC-MS analyses tentatively confirmed significant aroma profile differences among the rambutan seed oils.

To elucidate differences between sample groups and identify potential differential volatile compounds, OPLS-DA models were constructed for pairwise comparisons, namely BR-4 vs. BR-5, BR-4 vs. BR-7, and BR-5 vs. BR-7 (18). Significant separation was observed in all paired groups, as depicted in the score plot (Supplementary Figures S2A,E,I), indicating substantial aroma composition variations. Additionally, Supplementary Table S2 outlines classification parameters (*R*^2^X_(*cum*)_, *R*^2^Y_(*cum*)_, and Q^2^_(*cum*)_) for all OPLS-DA models, all exceeding 0.5, affirming model validity. High predictive capability was further supported by 200 cross-validation trials (Supplementary Figures S2B,F,J) (16). The S-plots visually represent volatile compounds projections, with blue dots denoting compounds with variable influence on projection (VIP) ≤ 1 and green triangles representing those with VIP > 1. A total of 17 (e.g., 2-octanol and pentyl 3-methylbutanoate), 10 (e.g., azetidine, 1,2-dimethyl-, and propanoic acid, ethenyl ester), and 12 (e.g., 2-aminocyanoacetamide and benzene, 1,3-dimethyl-) volatile compounds exhibited VIP values >1 (Supplementary Figures S2C,G,K), indicating their significant contribution to classification. These compounds dispersed both positively and negatively from the origin, with greater distances signifying higher classification contributions. Additionally, Supplementary Figures S2D,H,L highlight the top 10 differential volatiles based on VIP values for each pairwise group. In summary, differential volatiles in different varieties of rambutan seed oils were initially screened.

Fold change (FC) analysis is a statistical method frequently employed to identify differences between samples. To better visualize variations in aroma substance content among different rambutan seed oil varieties, FC values were calculated between groups, with criteria set at FC > 2 or < 0.5. The dynamic distribution plots clearly show the changes in the content of all aroma substances between the two groups (Supplementary Figures S3A,C,E), with the top 10 up- and down-regulated components emphasized. For BR-4 vs. BR-5, 2,3,4-Trimethyl-1-pentanol and benzene, 1-ethyl-2,4-dimethyl- were the most significantly differential compounds for up- and down-regulation, respectively. Ethanol was notably up-regulated, while oxalic acid, butyl cyclobutyl ester was down-regulated in BR-4 vs. BR-7. In BR-5 vs. BR-7, benzene, 1-ethyl-2,4-dimethyl- showed the highest FC value, with pentyl 3-methylbutanoate having the lowest. Subsequently, the *t*-test-derived *p*-value, with a threshold of *p* < 0.05, was utilized as an additional screening indicator. Volcano plots, plotting Log_2_FC values against -Log_10_P, were employed to visualize differences in volatile compound content between group pairs (Supplementary Figures S3B,D,F). Each dot represents a volatile compound: grey indicates no significant difference, red indicates up-regulation, and blue indicates down-regulation. Employing screening criteria of FC > 2 or < 0.5 and *p* < 0.05, we identified 100 differential volatiles in BR-4 vs. BR-5. Among these, 61 substances (e.g., tetradecanoic acid, 10,13-dimethyl-, methyl ester; 2,3,4-Trimethyl-1-pentanol, etc.) were significantly up-regulated, while 39 substances (e.g., benzene, 1-ethyl-2,4-dimethyl-; propiophenone, 2′-methyl-, etc.) were significantly down-regulated (Supplementary Figure S3B). In B5vsB7, 86 differential volatile compounds were screened. Of these, 49 compounds (e.g., ethanol, etc.) were down-regulated and 37 compounds (e.g., oxalic acid, butyl cyclobutyl ester, etc.) were up-regulated (Supplementary Figure S3D). Furthermore, ninety-one differential substances were detected in the comparison of BR-5 and BR-7, with 51 (e.g., benzaldehyde, 4-(1-phenyl-2-propenyloxy)-, etc.) up-regulated and 40 (e.g., pentyl 3-methylbutanoate, etc.) down-regulated (Supplementary Figure S3F).

**Figure 2:**
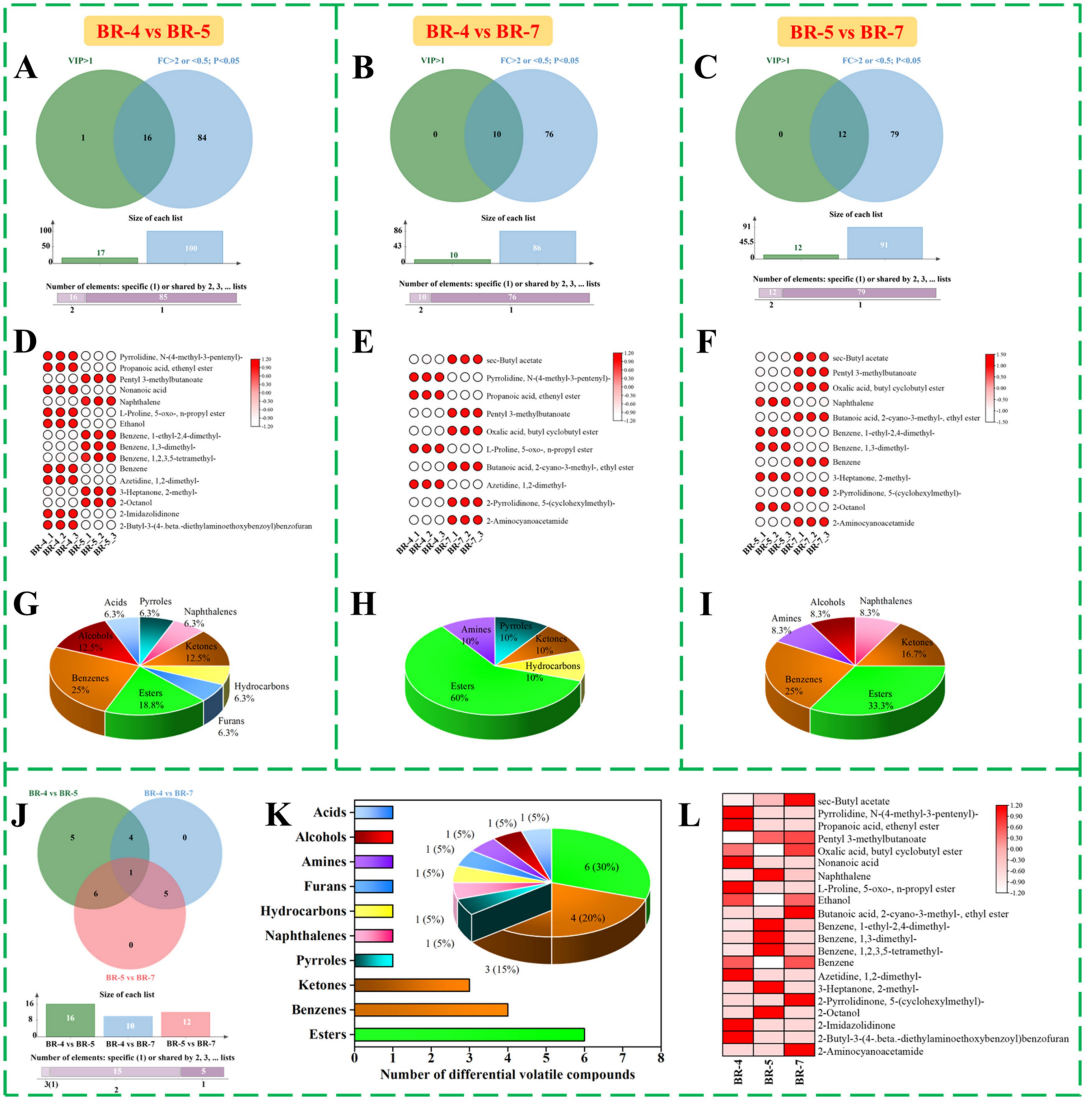
Screening and comparison of differential volatile compounds identified by GC-MS. Venn diagram of variable influence of projection (VIP) and fold change (FC), volatile compounds meeting the conditions were differential volatile compounds between BR-4 and BR-5 **(A)**, HR-4 vs. BR-7 **(B)**, BR-5 and BR-7 **(C)**. Heat map analysis and percentage of the categories of 16 differential volatile compounds in BR4 and BR-5 **(D,G)**, 10 differential volatile compounds in BR-4 vs. BR-7 **(E,H)**, and 12 differential volatile compounds in BR-5 vs. BR-7 **(F,I)**. Venn diagram of differential volatile compounds in BR-4 and BR-5, BR-4 vs. BR-7, and BR-5 vs. BR-7 **(J)**. Number and percentage of the categories of 21 key differential volatile compounds **(K)**. Heat map analysis of 21 key differential volatile compounds in rambutan seed oils **(L)**.

Following the outlined criteria (VIP > 1, FC > 2 or < 0.5, and *p* < 0.05), we identified 16 key differential volatiles in BR-4 vs. BR-5 (Supplementary Table S3). These included 1 pyrrole, 1 naphthalene, 2 ketones, 1 hydrocarbon, 1 furan, 3 esters, 4 benzenes, 2 alcohols, and 1 acid (Figures 2A,D,G). In the BR-4 and BR-7 comparisons, 10 differential aroma substances were screened (Supplementary Table S4), consisting of 1 pyrrole, 1 ketone, 1 hydrocarbon, 6 esters, and 1 amine (Figures 2B,E,H). Additionally, 12 differential volatiles, such as 2-octanol and oxalic acid, butyl cyclobutyl ester, were identified in BR-5 vs. BR-7 (Supplementary Table S5; Figures 2C,F,I). Integrating the results of the pairwise comparisons, we considered 21 volatiles as potential biologically significant markers (Supplementary Table S6). To gain further insights into the distribution of these aroma components, we conducted a cross-tabulation analysis (Figure 2J). Pentyl 3-methylbutanoate was the only compound identified in all pairwise comparisons, as depicted in the Venn diagram. Furthermore, four volatile compounds, including azetidine, 1,2-dimethyl-, and propanoic acid, ethenyl ester, appeared in both BR-4 vs. BR-5 and BR-4 vs. BR-7. Oxalic acid, 2-pyrrolidinone, 5-(cyclohexylmethyl)-, sec-butyl acetate, butyl cyclobutyl ester, butanoic acid, 2-cyano-3-methyl-, ethyl ester, and 2-aminocyanoacetamide were shared by BR-4 vs. BR-7 and BR-5 vs. BR-7. Six differential volatile compounds, mainly esters, were identified in both BR-4 vs. BR-5 and BR-5 vs. BR-7 (Figures 2K,L), indicating significant variation in their contents across varieties. Overall, these 21 aroma components could be used as potential biomarkers to distinguish between the seed oils extracted from the three rambutan varieties.

### 3.3 GC-IMS topographic plots of rambutan seed oils

GC-IMS combines the high separation performance of gas chromatography with the fast response and high sensitivity of ion mobility spectrometry. Notably, the different detection principles of MS and IMS lead to disparities in compound identification between GC-IMS and GC-MS (31). Hence, combining these techniques allows for a comprehensive analysis of food flavor attributes.

Volatile organic compounds from different rambutan seed oil varieties were analyzed using HS-GC-IMS. The resulting 3D topography visualizes the aroma composition, with the X-axis indicating measurement run time, the Y-axis representing drift time, and the Z-axis denoting peak intensity. The similar images with varying peak intensities suggest differences in aroma components among the rambutan seed oils (Supplementary Figure S4A). To better comprehend these differences, a top view of the three-dimensional topography was generated, depicting drift time on the horizontal axis and measurement run on the vertical axis (Supplementary Figure S4B). In the diagram, each point represents a volatile compound. Most signals occurred between 0 and 700 s with a drift time of 1.0 and 1.75 s (Supplementary Figure S4B). Furthermore, the volatile compound content in BR-4 served as the standard, with values from other groups subtracted accordingly (Supplementary Figure S4C). Color intensity reflects differences in volatile concentration: white for similar values, red for higher values, and blue for lower values. Evidently, significant differences exist in the volatile content among the rambutan seed oils. However, precise identification of flavor substances remains challenging at this stage.

### 3.4 Comparison of volatile compounds in rambutan seed oils based on HS-GC-IMS

**Figure 3:**
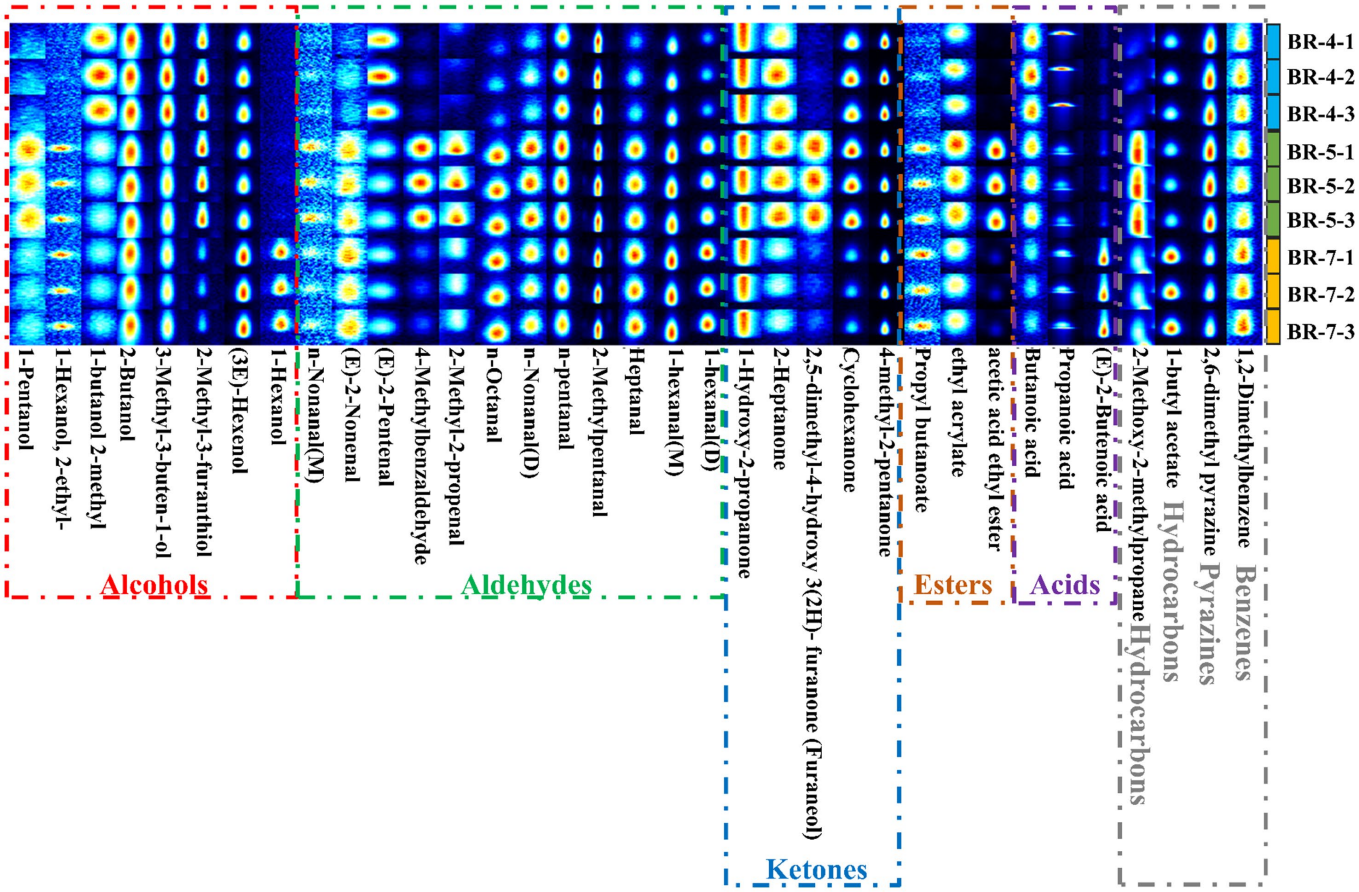
Gallery plot comparison of volatile compounds in seed oils extracted from three rambutan varieties.

To elucidate the variation in volatile compounds among different rambutan seed oil varieties, gallery plots were automatically generated using all analyzed signals (Figure 3). Each row represents a sample, and each column represents a volatile substance. The color scale illustrates the compound content level, with brighter colors indicating higher levels (13).

**Figure 4:**
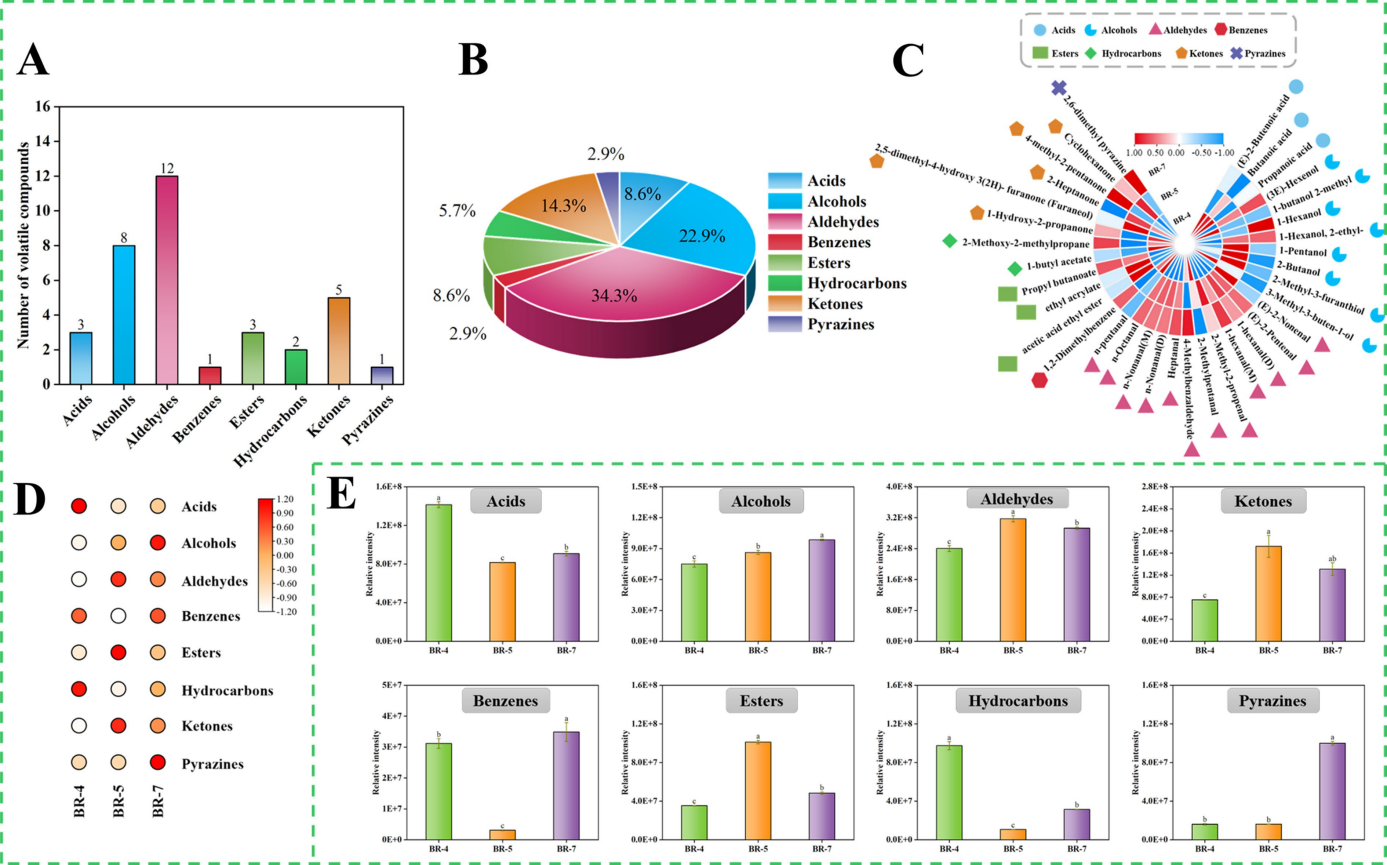
Overview of volatile compounds identified by GC-IMS in rambutan seed oils. Numbers **(A)** and Percentage **(B)** of volatile compounds identified in rambutan seed oils. Cluster heat map of all volatile compounds in rambutan seed oils **(C)**. Heat map of different volatile compound species in rambutan seed oils **(D)**. Intensity of different species of volatile compound in rambutan seed oils **(E)**. The different letters in the bar chart were shown as a significant difference between samples (*p* < 0.05).

A qualitative analysis of volatile compounds in rambutan seed oil was conducted, and the findings were presented in Supplementary Figure S5 and Supplementary Table S7. Thirty-five volatile compounds were identified via GC-IMS, encompassing 3 acids, 8 alcohols, 12 aldehydes, 1 benzene, 3 esters, 2 hydrocarbons, 5 ketones, and 1 pyrazine (Supplementary Table S7). Notably, certain compounds displayed double peaks, indicating the presence of both monomer and dimer. This occurrence can be attributed to compounds with high proton affinity, which can induce ion formation of dimers during drift tank traversal (12). Analysis of the GC-IMS data revealed that aldehydes were the predominant volatile compounds in rambutan seed oil, succeeded by alcohols, ketones, and acids (Figures 4A,B). Similar trends were observed in passion fruit seed oil (12) and *Trichosanthes kirilowii* seed oil (32). Additionally, a cluster heatmap was generated to illustrate the aroma profile of rambutan seed oils based on the 35 volatiles identified (Figure 4C). To effectively explore differences in volatile compounds among various rambutan seed oil varieties, the results were compared by summing the peak areas of volatiles within the same class, visualized as heatmaps (Figure 4D) and histograms (Figure 4E).

Aldehydes significantly influence food flavor due to their low odor threshold and high volatility. They primarily result from the oxidation of unsaturated fatty acids. Our study detected a total of 12 aldehydes. Aldehyde levels varied significantly among samples, with BR-5 exhibiting notably higher levels than other groups (Figure 4E). N-pentanal, (E)-2-nonenal, and 4-methylbenzaldehyde were the predominant aldehydes in BR-4, BR-5, and BR-7, respectively (Supplementary Table S7; Figure 4C). Interestingly, Ong et al. (33) reported that (E)-2-nonenal was a major contributor to the flavor of Jitlee rambutan fruits from Singapore. Additionally, (E)-2-nonenal was detected in green plum seeds (10). Recent research revealed that pentanal is the primary aldehyde in passion fruit seeds (12). Moreover, 4-methylbenzaldehyde has been used in perfume and fragrance production due to its potent aroma. Additionally, 2-methylpentanal, 1-hexanal (M), n-octanal, 1-hexanal (D), n-nonanal (M), n-nonanal (D), and (E)-2-nonenal were significant contributors to the flavor profile of rambutan seed oil. Most of these aldehydes result from the oxidation of fatty acids. For example, hexanal, characterized by a green, leafy, or woody aroma, primarily results from the decomposition of linoleic acid-13-COOH through *β*-homogenous cracking, possibly involving the generation of 1-hexenyl radical and hydroxyl group through β-homogenous cracking of linoleic acid-12-COO (32). Octanal and nonanal, oxidation products of linoleic and alpha-linolenic acids, primarily emit green and fatty aromas (34). A previous study also identified nonanal in rambutan seed lipids (8). Overall, different aldehydes imparted distinct flavor profiles to the three rambutan seed oils. Zeng et al. (35) had also reported that aldehydes are also the main volatile substances of Camellia oleifera oil (36), and Sun et al. (37) also concluded that aldehydes were considered to be the main contributors to the aroma of Blue honeysuckle seeds (35).

Pyrazines are typically desirable in food products due to their contribution to flavors like nutty and bakery aromas. These compounds are commonly linked to non-enzymatic browning reactions. Our study identified only one pyrazine, specifically 2,6-dimethyl pyrazine, in rambutan seed oil via GC-IMS. The volatile level in BR-7 was approximately six times higher (*p* < 0.05) than that in BR-4 and BR-5, as depicted in Figure 4E. Analogous findings were reported by Khairy et al. (8), identifying pyrazine, 2-ethyl-6-methyl- and pyrazine, 2,6-dimethyl- as the main pyrazines in rambutan seed oil from Malaysia. Additionally, they reported eight other pyrazines, including pyrazine, 3,5-diethyl-2-methyl- (18). These differences may stem from variations in cultivars, growing environments, and geographical origins.

Acids mainly result from triglyceride decomposition and free fatty acid oxidation, enhancing the oily flavor of rambutan seed oil. Our study detected three acids: two saturated (butanoic acid and propanoic acid) and one unsaturated ((E)-2-butenoic acid) (Supplementary Table S7). Similarly, butanoic acid and propanoic acid were also observed in rapeseed oil (37) and passion fruit seed oil (12). While previous studies did not detect propanoic acid in rambutan seed oil, its derivatives propanoic acid, 2-methyl-, butyl ester, and propanoic acid, 2-methyl- were identified (8). Importantly, the levels of (E)-2-butenoic acid, butanoic acid, and propanoic acid were markedly higher in BR-4 compared to BR-5 and BR-7 (Figure 4C). In addition to the discussed volatile compounds, GC-IMS detected alcohols (8), ketones (5), esters (3), hydrocarbons (2), and benzenes (1) in rambutan seed oils (Supplementary Table S7). Eight alcohols were identified, including (3E)-hexenol, 1-butanol 2-methyl, 1-hexanol, 1-hexanol, 2-ethyl-, 1-pentanol, 2-butanol, 2-methyl-3-furanthiol, and 3-methyl-3-buten-1-ol. One prior study also found 1-hexanol and 1-pentanol in Malaysian rambutan seed oil (8). To the best of our knowledge, (3E)-hexenol, 1-butanol 2-methyl, 1-hexanol, 2-ethyl-, 2-butanol, 2-methyl-3-furanthiol, and 3-methyl-3-buten-1-ol are reported in rambutan seed oil for the first time. Furthermore, these volatiles exhibited significant differences among the three types of rambutan seed oils. Concerning ketones and esters, BR-5 exhibited significantly higher levels compared to BR-4 and BR-7. Generally, the aroma profile of rambutan seed oil varies depending on the variety, and these aroma compounds contribute to the processing potential of the oil.

### 3.5 Multivariate statistical analysis of volatile compounds in rambutan seed oils based on HS-GC-IMS

**Figure 5:**
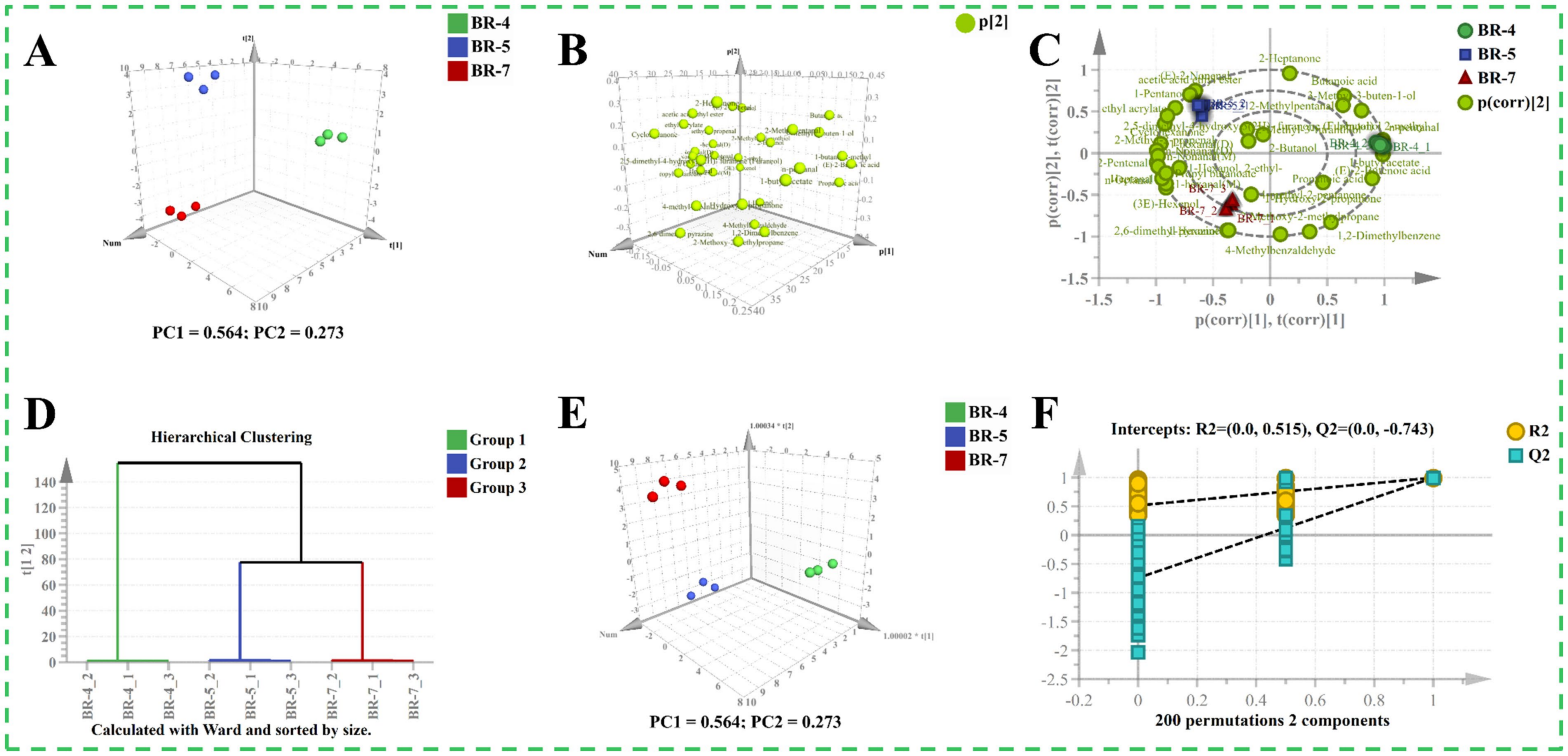
Multivariate statistical analysis of volatile compounds identified by GC-IMS of rambutan seed oils. Score plot of PCA model **(A)**. Loading plot **(B)**. Biplot of PCA model **(C)**. Hierarchical clustering analysis plot **(D)**. Score plot of OPLS-DA model **(E)**. 200 times permutation test plot **(F)**.

To preliminarily distinguish between different varieties of rambutan seed oils, we analyzed the GC-IMS data using a PCA model (10). The 3D score plot of the PCA depicted well-defined categorization of all samples into three distinct groups (Figure 5A), indicating significant differences in aroma profiles. The first two components accounted for the majority of the total variation, with components 1 and 2 explaining 56.40 and 27.30%, respectively. Loading plots and biplots were provided as supplementary information for the PCA scoring plots (Figures 5B,C). For particular example, the contribution of (E)-2-butenoic acid was higher in BR-4 compared to BR-5 and BR-7. There was a greater contribution of 1-pentanol in BR-5 than in the other groups. Additionally, 1-hexanol predominantly differentiated BR-7 from BR-4 and BR-5. Subsequently, hierarchical cluster analysis (HCA) was employed to identify differences and similarities among the samples. Samples were classified into three groups based on the dataset’s characteristics (Figure 5D). Clearly, Group 2 and Group 3 were grouped together, indicating that the aroma profiles of BR-5 and BR-7 are more similar to each other than to BR-4. Further, an OPLS-DA model in supervised mode was constructed to extract sample clustering information and identify potential aroma markers between groups (18). Similar to the PCA model, the samples formed three distinct clusters, but they exhibited closer proximity (Figure 5E). The *R*^2^X_(*cum*)_, *R*^2^Y_(*cum*)_, and Q^2^ values of the OPLS-DA model were 0.837, 0.996, and 0.989, respectively, indicating the model’s stability and predictability. Furthermore, the results of a 200-fold cross-validation test demonstrated the rationality and validity of the model (Figure 5F).

OPLS-DA models were utilized for pairwise comparisons to further analyze the dataset obtained from GC-IMS (16). Supplementary Figures S6A,D,G illustrate clear separation in all pairwise comparisons, indicating significant differences in volatiles between each group. In the S-plots, compounds with VIP values exceeding 1 were highlighted in orange (Supplementary Figures S6B,E,H). Based on the screening criterion VIP > 1, 11 varieties (including n-pentanal and acetic acid ethyl ester), 10 varieties (including (E)-2-butenoic acid and 4-methylbenzaldehyde), and 8 varieties (including 4-methyl-2-pentanone and 1,2-dimethylbenzene) were identified in pairwise comparisons between BR-4 vs. BR-5, BR-4 vs. BR-7, and BR-5 vs. BR-7, respectively. The validation parameters of the model were presented in Supplementary Table S8 and Supplementary Figures S6C,F,I, confirming the models’ strong reliability.

To enhance the accuracy of screening potential differential aroma substances, FC and *p* values were utilized as screening thresholds (18). Dynamic distribution plots (Supplementary Figures S7A,C,E) display the FC values for all volatile compounds, with the top five most significantly altered molecules highlighted in red (up-regulated) and blue (down-regulated). The volcano plot (Supplementary Figures S7B,D,F) illustrates that a total of 16 (6 up-regulated and 10 down-regulated), 14 (3 up-regulated and 11 down-regulated), and 9 (3 up-regulated and 6 down-regulated) differentially volatile compounds were identified in BR-4 vs. BR-5, BR-4 vs. BR-7, and BR-5 vs. BR-7, respectively, using the filtering criteria of FC > 2 or < 0.5 and *p* < 0.05.

The screening criteria, including VIP > 1, FC > 2 or < 0.5, and *p* < 0.05, were combined to identify key differential compounds, depicted in Venn diagrams (Supplementary Figures S8A,D,G; Supplementary Tables S9–S11). In the comparison between BR-4 and BR-5, 11 potentially differential volatile compounds were identified. Among these 11 compounds, n-nonanal (D), cyclohexanone, acetic acid ethyl ester, 2-methyl-2-propenal, (E)-2-pentenal, and (E)-2-nonenal were found in significantly lower amounts in BR-4 compared to BR-7. Conversely, the levels of *n* pentanal, 4-methylbenzaldehyde, 1-butyl acetate, 1,2-dimethylbenzene, and (E)-2-butenoic acid were significantly higher in BR-4 than in BR-7 (Supplementary Figures S8B,C). Eight differential volatiles were identified in the comparison between BR-4 and BR-7. Among these, three compounds (n-pentanal, 1-butyl acetate, and (E)-2-butenoic acid) were significantly higher in BR-4 than in BR-7, while the remaining five volatile compounds, including heptanal, cyclohexanone, 2,6-dimethyl pyrazine, (E)-2-pentenal, and (3E)-hexenol, were significantly lower in BR-4 than in BR-7 (Supplementary Figures S8E,F). In the pairwise comparisons between BR-5 and BR-7, seven potential differential aroma components were identified. Cyclohexanone, acetic acid ethyl ester, and (e)-2-nonenal were found at significantly higher levels in BR-5 compared to BR-7. By contrast, BR-5 exhibited significantly lower levels of 4-methylbenzaldehyde, 2-methoxy-2-methylpropane, 2,6-dimethyl pyrazine, and 1,2-dimethylbenzene than BR-7 (Supplementary Figures S8H,I).

In conclusion, through integrating the results of pairwise comparisons, a total of 15 volatiles were identified as potential biologically significant aroma markers (Supplementary Table S12). Among these, two compounds (n-nonanal (D) and 2-methyl-2-propenal) were exclusively identified in BR-4 vs. BR-5, two varieties (heptanal and (3E)-hexenol) in BR-4 vs. BR-7, and one compound (2-Methoxy-2-methylpropane) in BR-5 vs. BR-7, respectively (Supplementary Figure S9A). Heat map was utilized to illustrate the variations in the levels of these 15 key differential volatiles, with the highest concentrations predominantly observed in BR-7 (Supplementary Figure S9B). Interestingly, the majority of these volatile compounds were classified as aldehydes (Supplementary Figure S9C), suggesting their significant contribution to the flavor profile of rambutan seed oil.

### 3.6 Relationships between volatile compounds

**Figure 6:**
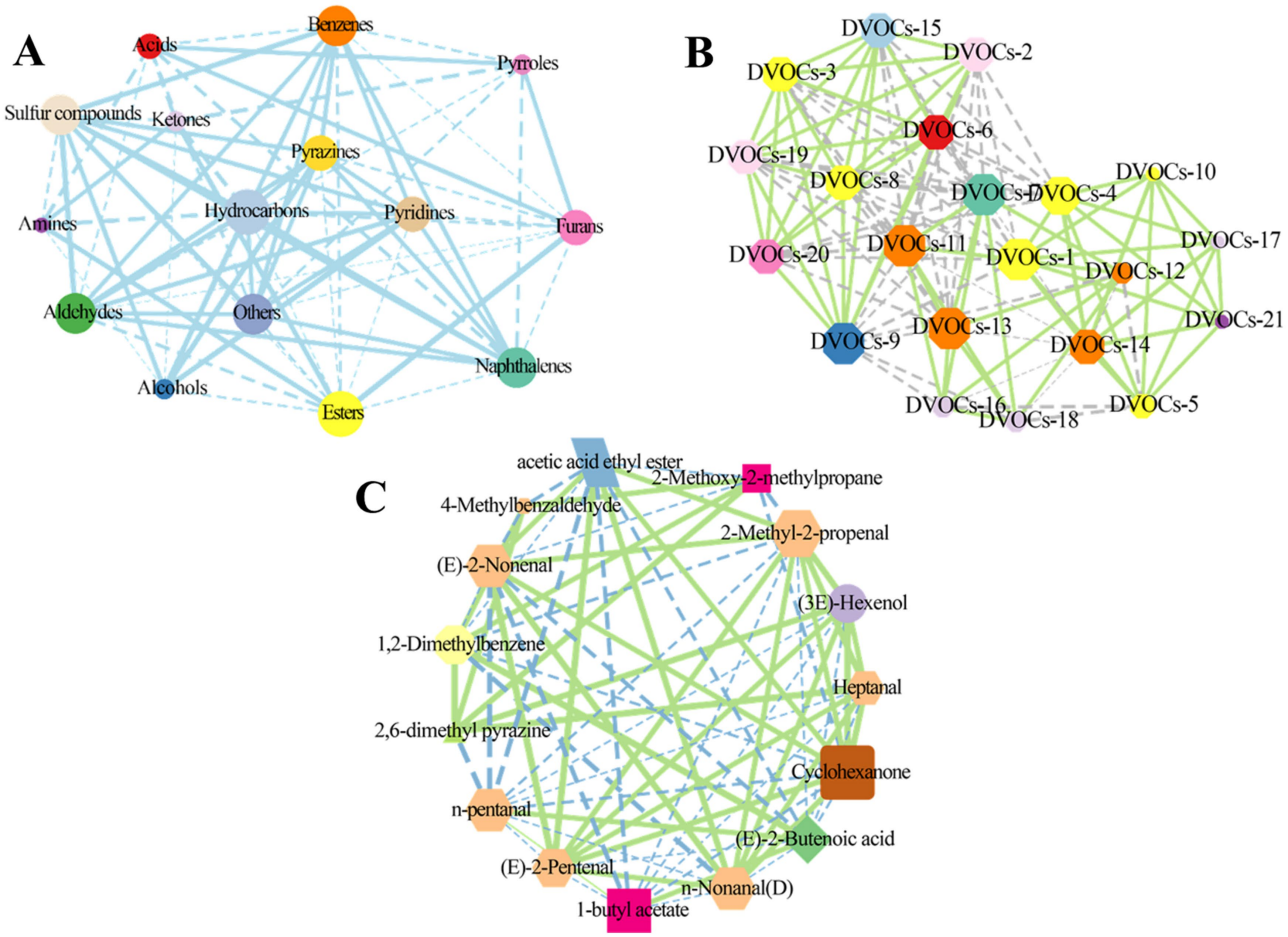
Correlation among volatile compounds. Pearson’s correlation network diagrams for the categories of volatile compounds **(A)** and differential volatile compounds **(B)** characterized by CC-MS, The names of the differential volatile compounds corresponding to the serial numbers are shown in Supplementary Table S6. Pearson’s correlation network diagrams for the differential volatile compounds identified by GC-IMS **(C)**. In the correlation network diagram, the solid line represents a positive correlation and the dashed line represents a negative correlation.

Pearson’s correlation analysis was employed to investigate the relationships among aroma components (32). The correlation coefficient threshold was set to |r| > 0.5. Dashed lines represent a negative correlation, while solid lines represent positive correlation. In Figure 6A, esters exhibited positive correlations with ketones (r = 0.754), furans (r = 0.521), and amines (r = 0.768), while showing negative correlations with aldehydes (r = −0.624), benzenes (r = −0.745), hydrocarbons (r = −0.947), and alcohols (r = −0.827). Aldehydes demonstrated significant correlations with 11 other families of aroma compounds, including acids, hydrocarbons, and pyrazines, among others, with most exhibiting positive correlations. Except for esters, ketones, and amines, sulfur compounds displayed significant positive correlations with seven other categories of aroma components, including esters, pyridines, pyrazines, naphthalenes, hydrocarbons, benzenes, aldehydes, and alcohols. Furans displayed significant negative correlations with benzenes (r = −0.955), aldehydes (r = −0.990), and hydrocarbons (r = −0.759), while exhibiting positive correlations with esters (r = 0.521) and acids (r = 0.912).

Further, we analyzed the correlation between the differentially volatile compounds screened by GC-MS analysis. Clearly, there were complex correlations between the 21 volatile compounds. For instance, ethanol displayed negative correlation with sec-Butyl acetate (r = −0.540) and naphthalene (r = −0.693), and positive correlation with pyrrolidine, N-(4-methyl-3-pentenyl)- (r = 0.990), propanoic acid, ethenyl ester (r = 0.990), L-proline, 5-oxo-, n-propyl ester (r = 0.991), and nonanoic acid (r = 0.987). Propanoic acid, ethenyl ester was negatively correlated with sec-butyl acetate (r = −0.648), naphthalene (r = −0.591), pentyl 3-methylbutanoate (r = −0.598) and positively correlated with nonanoic acid (r = 0.997), 2-imidazolidinone (r = 0.999), and azetidine, 1,2-dimethyl- (r = 0.997) (Figure 6B).

Moreover, correlations between differential volatile compounds obtained by GC-IMS analysis were also analyzed. In Figure 6C, N-pentanal exhibited positive correlations with (E)-2-butenoic acid (r = 0.981) and 1-butyl acetate (r = 0.996), while showing negative correlations with cyclohexanone (r = −0.809), acetic acid ethyl ester (r = −0.578), 2,6-dimethyl pyrazine (r = −0.515), n-nonanal (D) (r = −0.980), and heptanal (r = −0.975). Except for 4-methylbenzaldehyde, 1,2-dimethylbenzene, and cyclohexanone, 1-butyl acetate exhibited correlations with the remaining 10 differential volatiles, predominantly negative. Cyclohexanone displayed correlations with 12 differential volatiles, including acetic acid ethyl ester and (3E)-hexenol, among others, with the majority being positive correlations. These results confirm that the aroma profiles of rambutan seed oils are influenced by various volatile compounds and that significant differences exist among different varieties of rambutan seed oils.

## 4 Conclusion

Rambutan seed oil, emerging as a novel vegetable oil resource, exhibits promising potential for applications in the food, pharmaceutical, and cosmetic industries. The integrated approach of HS-SPME-GC-MS and HS-GC-IMS facilitated a comprehensive elucidation of the aroma profile of rambutan seed oils. Using GC-MS analysis, 135 volatile compounds, mainly hydrocarbons, and esters, were identified from rambutan seed oils. GC-IMS analysis detected 35 volatile compounds, primarily aldehydes and alcohols, in rambutan seed oils. Multivariate statistical analysis unveiled notable variations in the aroma profiles among the three types of rambutan seed oils. BR-5 exhibited significantly higher levels of aldehydes and hydrocarbons compared to BR-4 and BR-7. In BR-4, BR-5, and BR-7, n-pentanal, (E)-2-nonenal, and 4-methylbenzaldehyde emerged as the predominant aldehydes, respectively. Compared to the other two groups, BR-7 exhibited notably elevated levels of esters and ketones. The sole pyrazine detected, 2,6-dimethyl pyrazine, was roughly six times more prevalent in BR-7 than in BR-4 and BR-5. BR-4 and BR-5 exhibited the presence of sulfur-containing compounds, whereas they were absent in BR-7. Furthermore, 21 and 15 differentially volatile compounds were extracted from the datasets obtained via GC-MS and GC-IMS, respectively, utilizing the screening criteria of VIP > 1, FC > 2 or < 0.5, and *p* < 0.05. These differential volatile compounds consist predominantly of esters and aldehydes, contributing to the development of floral, fruity, green, and fatty aromas. Correlation analysis revealed intricate relationships among the differential volatile compounds. Esters exhibited positive correlations with ketones and furans, while showing negative correlations with aldehydes, hydrocarbons, and alcohols. These findings offer theoretical insights into assessing the flavor profile and harnessing the potential of rambutan seed oil. Further research is required to explore the correlation between aroma profiles and different processing technology and processing degree. Our experimental results laid the foundation for the subsequent discussion on the edibleness and sensory evaluation of rambutan seed oil.

## Data Availability

The original contributions presented in the study are included in the article/Supplementary material, further inquiries can be directed to the corresponding author.
