# Supplementary material for: Comprehensive characterization and comparison of aroma profiles of rambutan seed oils using GC-MS and GC-IMS combined with chemometrics
Source: Front Nutr. 2024 Oct 28;11:1486368. doi: 10.3389/fnut.2024.1486368 (PMC11552487; doi:10.3389/fnut.2024.1486368)
Supplement: Supplementary file 1 [file Data_Sheet_1.docx]

**Figure Captions**

**Fig. S1.** Multivariate statistical analysis of volatile compounds identified by GC-MS of rambutan seed oils. Score plot of PCA model (A). Loading plot (B). Biplot of PCA model (C). Hierarchical clustering analysis plot (D). Score plot of OPLS-DA model (E). 200 times permutation test plot (F).

**Fig. S2.** Multivariate statistical analysis of volatile compounds identified by GC-MS of pairwise groups. Score plots of OPLS-DA model for BR-4 vs BR-5, BR-4 vs BR-7, and BR-5 vs BR-7 (A, E, I). 200 times permutation test plots for BR-4 vs BR-5, BR-4 vs BR-7, and BR-5 vs BR-7 (B, F, J). S-plots of OPLS-DA model for BR-4 vs BR-5, BR-4 vs BR-7, and BR-5 vs BR-7 (C, G, K). Top 10 compounds with highest VIP scores of BR-4 vs BR-5, BR-4 vs BR-7, and BR-5 vs BR-7 (D, H, L).

**Fig. S3.** Variation of volatile compounds identified by GC-MS in pairwise comparison of rambutan seed oils. Dynamic distribution diagram of volatile compound content difference of BR-4 vs BR-5 (A), BR-4 vs BR-7 (C), and BR-5 vs BR-7 (E). Volcano plot of volatile compounds in BR-4 vs BR-5 (B), BR-4 vs BR-7 (D), and BR-5 vs BR-7 (F).

**Fig. S4.** GC-IMS spectrogram. 3D topographic plot of volatile compound profiles of rambutan seed oils (A). The two-dimensional topographic plot (B). Comparison of the difference spectrum of volatile compounds (C). With BR-4 as the reference, the signal peak in BR-4 is subtracted from the rest of the spectra and the difference in the spectra of the two samples was obtained. The blue color means that the substance is lower than BR-4 and the red dot means that the substance is higher than BR-4.

**Fig. S5.** Topographic plots of GC-IMS spectra with individual volatile compound identification marked 1–35. GC-IMS spectra of BR-4 (A), BR-5 (B), and BR-7 (C).

**Fig. S6.** Multivariate statistical analysis of volatile compounds identified by GC-IMS of pairwise groups. Score plots of OPLS-DA model for BR-4 vs BR-5, BR-4 vs BR-7, and BR-5 vs BR-7 (A, D, G). S-plots of OPLS-DA model for BR-4 vs BR-5, BR-4 vs BR-7, and BR-5 vs BR-7 (B, E, H). 200 times permutation test plots for BR-4 vs BR-5, BR-4 vs BR-7, and BR-5 vs BR-7 (C, F, I).

**Fig. S7.** Variation of volatile compounds identified by GC-IMS in pairwise comparison of rambutan seed oils. Dynamic distribution diagram of volatile compound content difference of BR-4 vs BR-5 (A), BR-4 vs BR-7 (C), and BR-5 vs BR-7 (E). Volcano plot of volatile compounds in BR-4 vs BR-5 (B), BR-4 vs BR-7 (D), and BR-5 vs BR-7 (F).

**Fig. S8.** Screening and comparison of differential volatile compounds identified by GC-IMS. Venn diagram of variable influence of projection (VIP) and fold change (FC), volatile compounds meeting the conditions were differential volatile compounds between BR-4 and BR-5 (A), BR-4 vs BR-7 (D), BR-5 and BR-7 (G). Heat map analysis and percentage of the categories of 11 differential volatile compounds in BR-4 and BR-5 (B, C), 8 differential volatile compounds in BR-4 vs BR-7 (E, F), and 7 differential volatile compounds in BR-5 vs BR-7 (H, I).

**Fig. S9.** Venn diagram of differential volatile compounds identified by GC-IMS in BR-4 and BR-5, BR-4 vs BR-7, and BR-5 vs BR-7 (A). Heat map analysis of 15 key differential volatile compounds in rambutan seed oils (B). Number and percentage of the categories of 21 key differential volatile compounds (C).

**Table Captions**

**Table S1.** Volatile compounds characterized in rambutan seed oils using GC-MS.

**Table S2.** The statistical parameters of PCA and OPLS-DA modes of oil samples from three rambutan seed (GC-MS).

**Table S3.** Differential volatile compounds identified by GC-MS in the group of BR-4 vs BR-5.

**Table S4.** Differential volatile compounds identified by GC-MS in the group of BR-4 vs BR-7.

**Table S5.** Differential volatile compounds identified by GC-MS in the group of BR-5 vs BR-7.

**Table S6.** Differential volatile compounds identified by GC-MS in rambutan seed oils.

**Table S7.** Volatile compounds characterized in rambutan seed oils using GC-IMS.

**Table S8.** The statistical parameters of PCA and OPLS-DA modes of oil samples from three rambutan seed (GC-IMS).

**Table S9.** Differential volatile compounds identified using GC-IMS in the group of BR-4 vs BR-5.

**Table S10.** Differential volatile compounds identified using GC-IMS in the group of BR-4 vs BR-7.

**Table S11.** Differential volatile compounds identified using GC-IMS in the group of BR-5 vs BR-7.

**Table S12.** Differential volatile compounds identified using GC-IMS in rambutan seed oils.


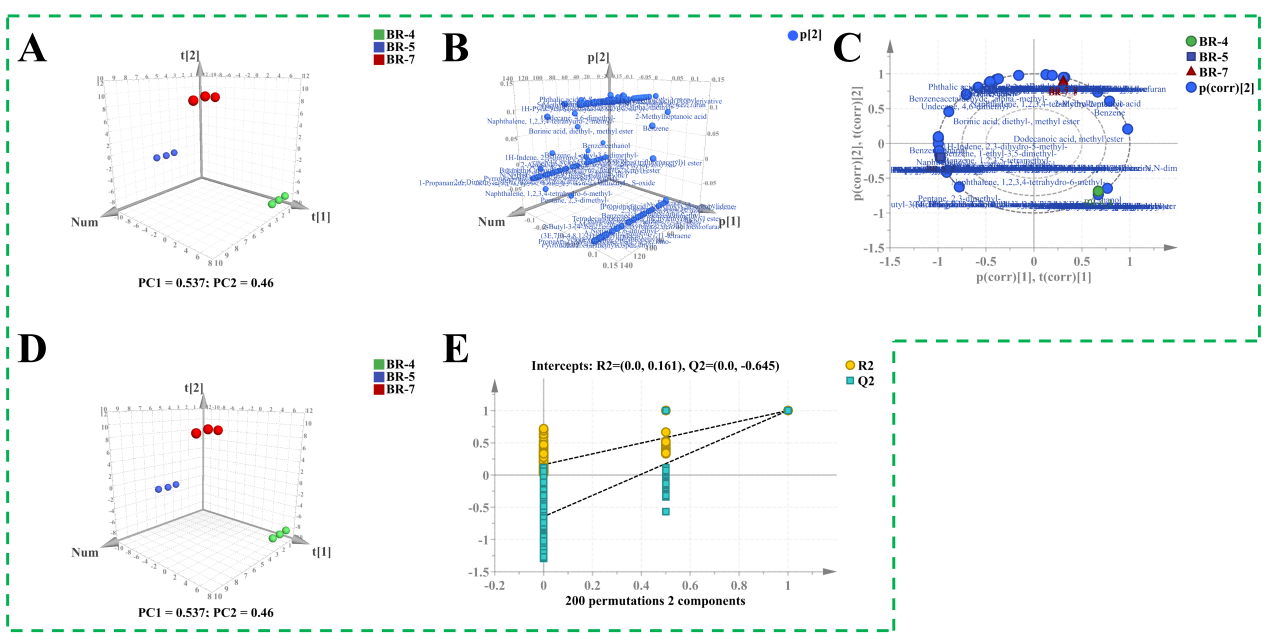


**Fig. S1.**


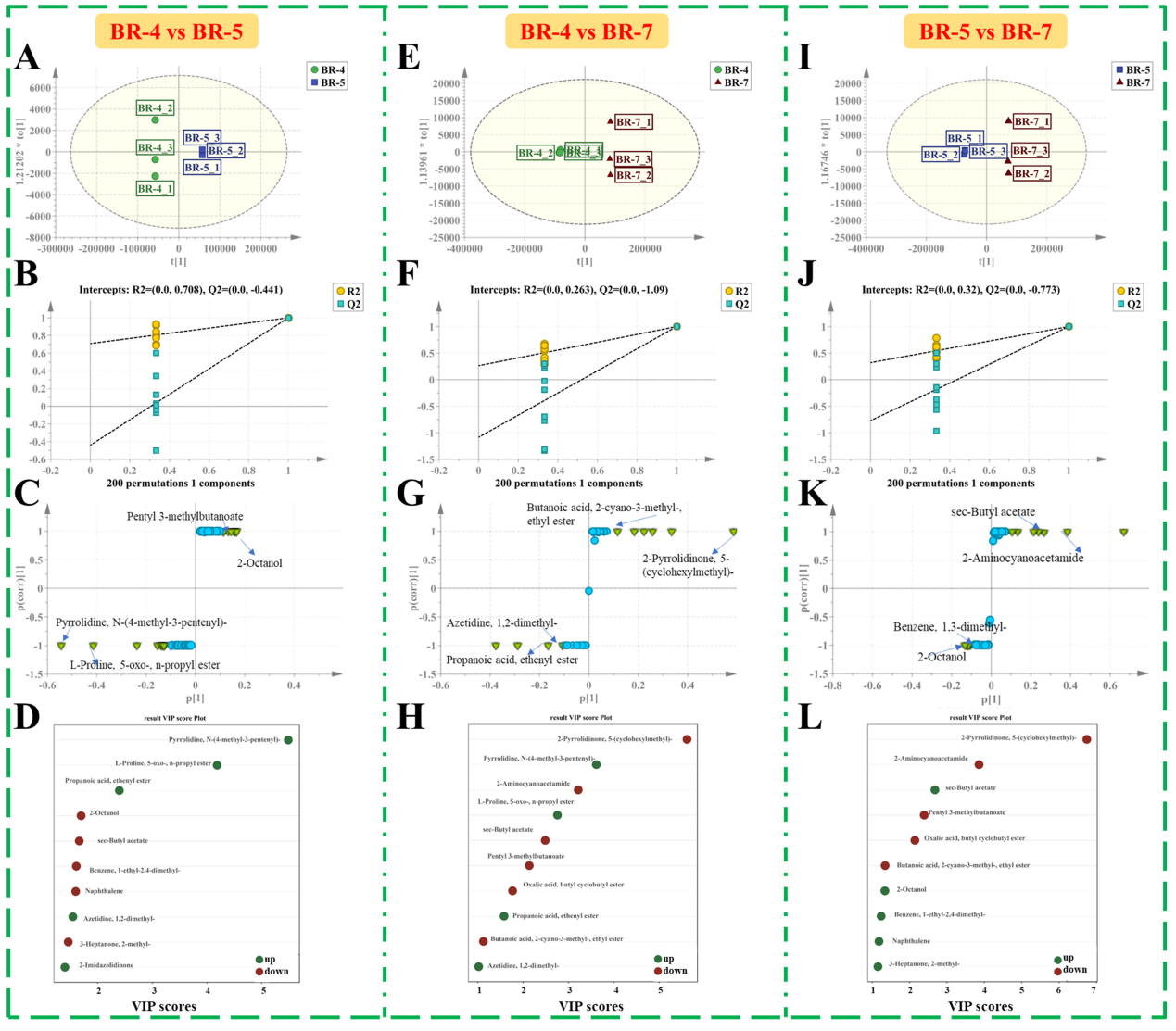


**Fig. S2.**

**
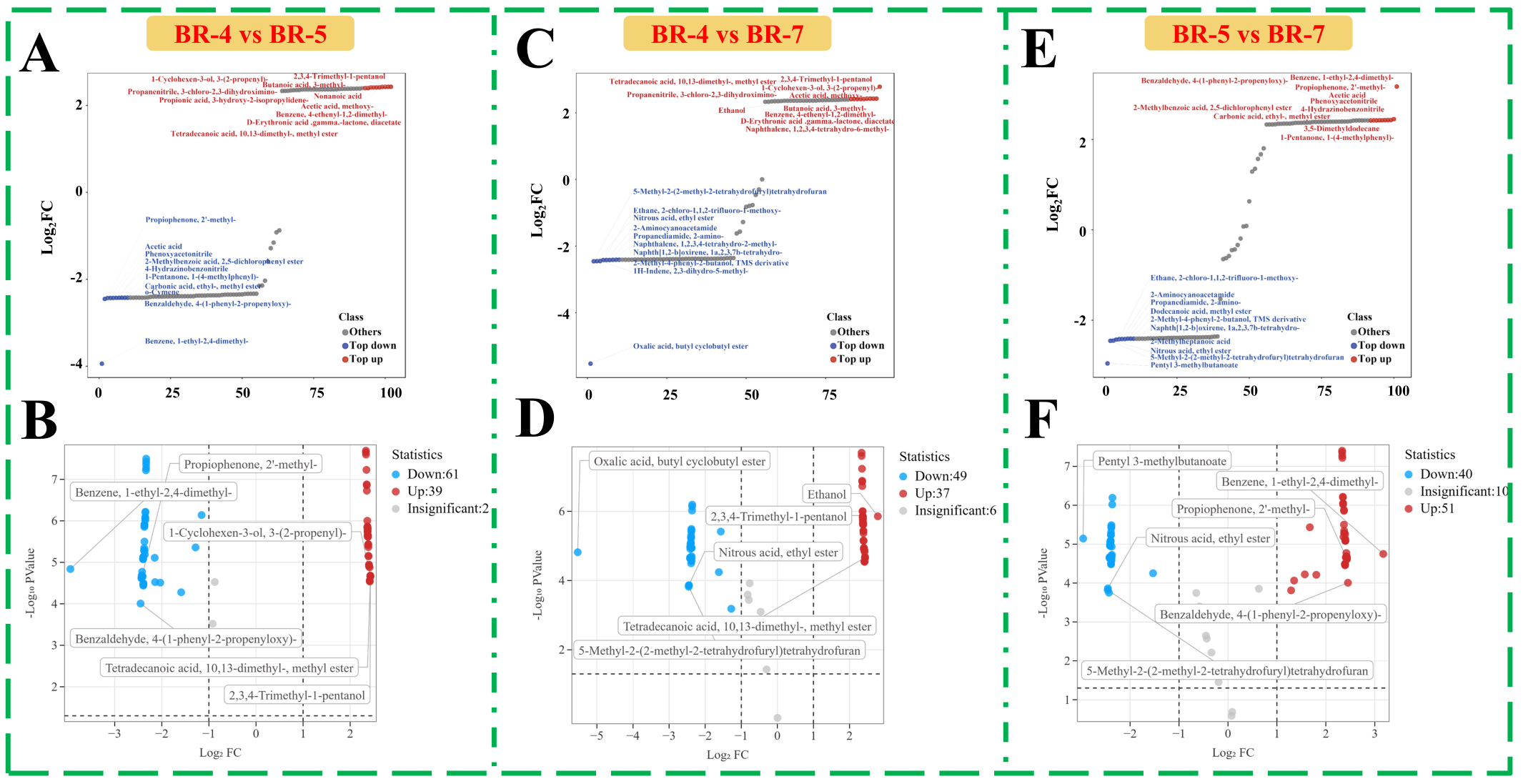
**

**Fig. S3.**

**
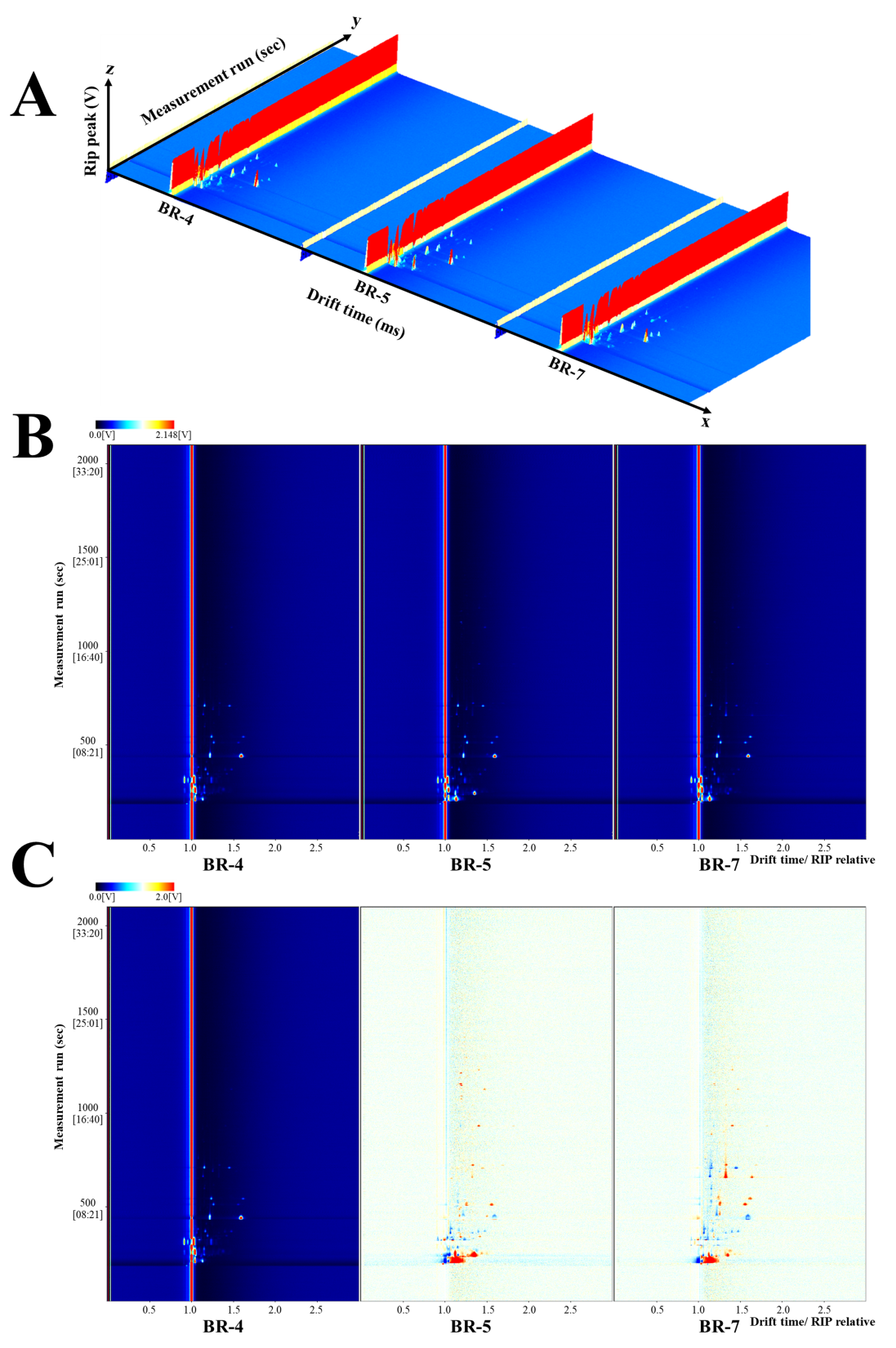
**

**Fig. S4.**

**
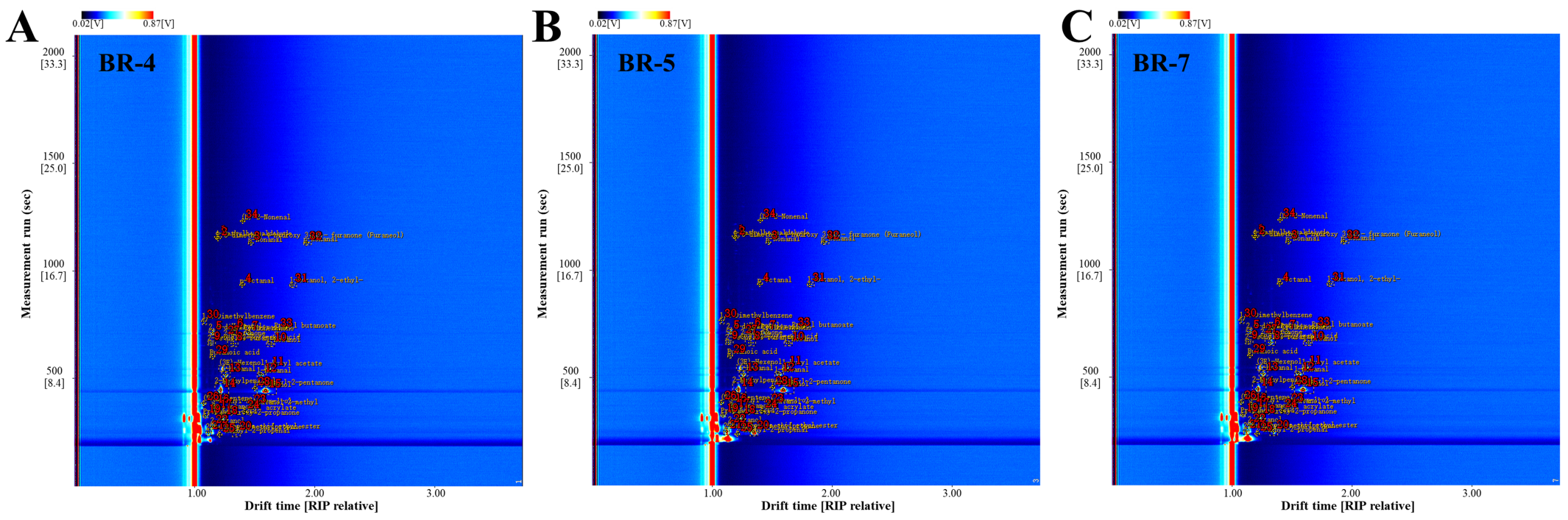
**

**Fig. S5.**

**
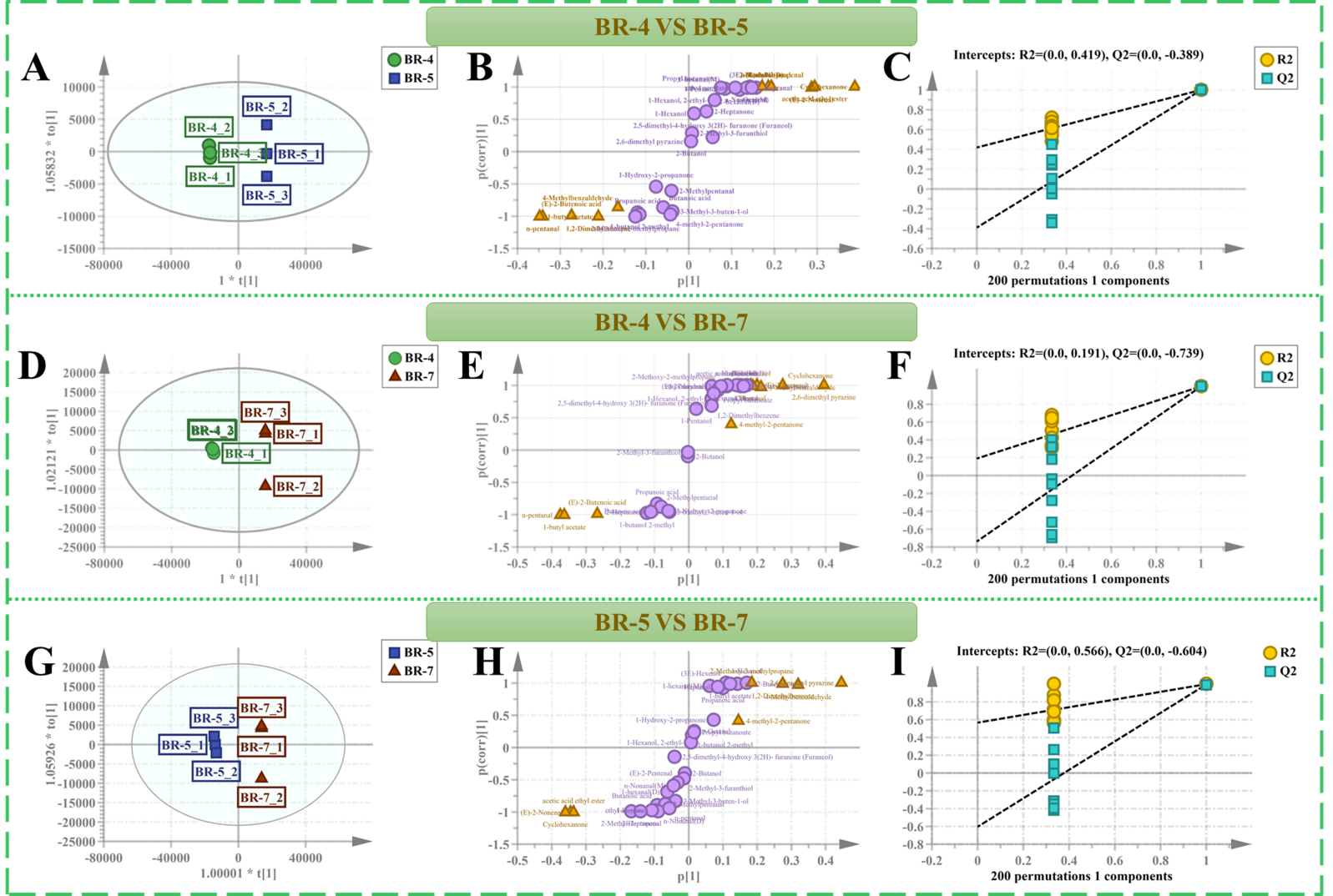
**

**Fig. S6.**

**
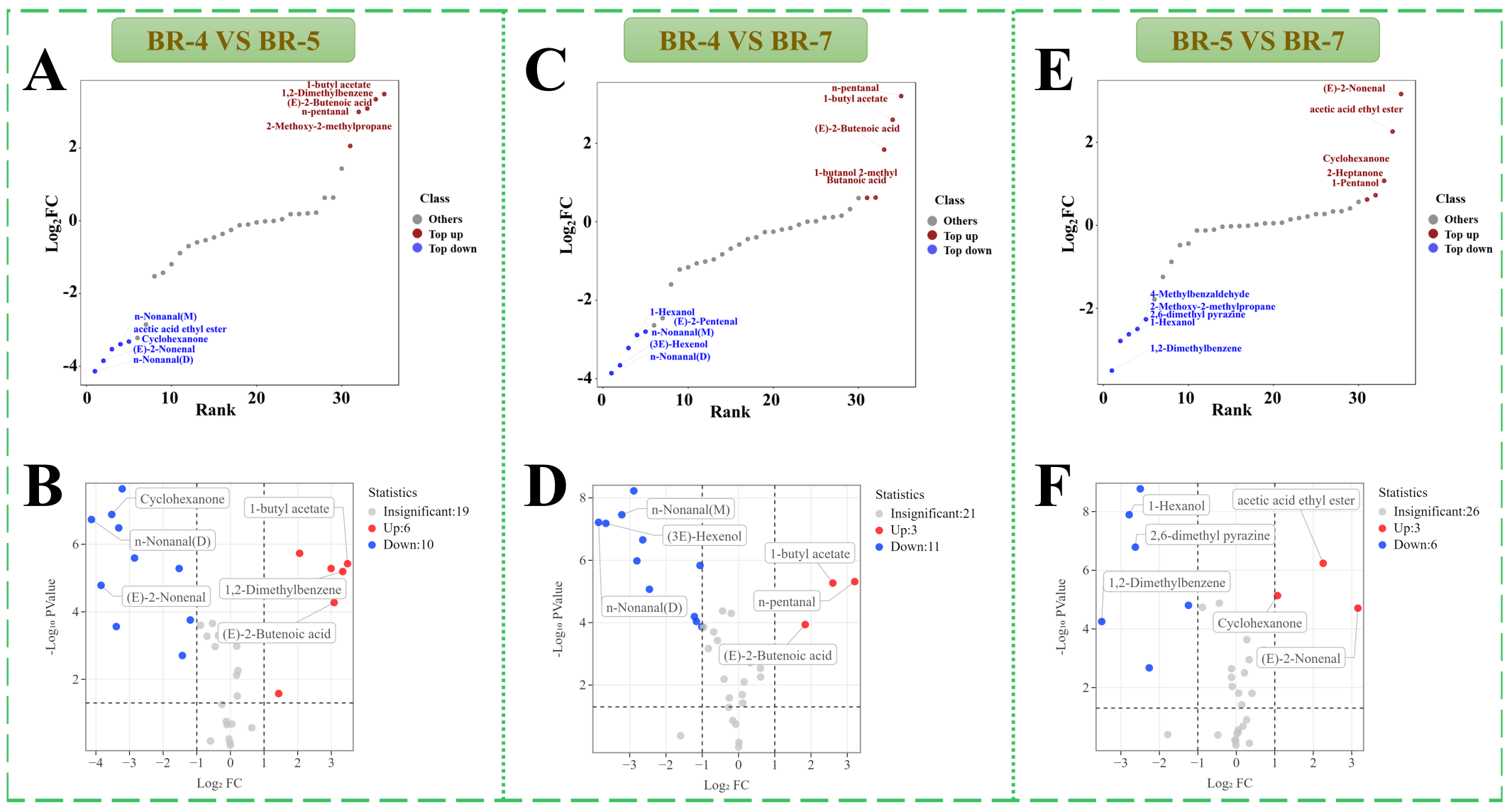
**

**Fig. S7.**

**
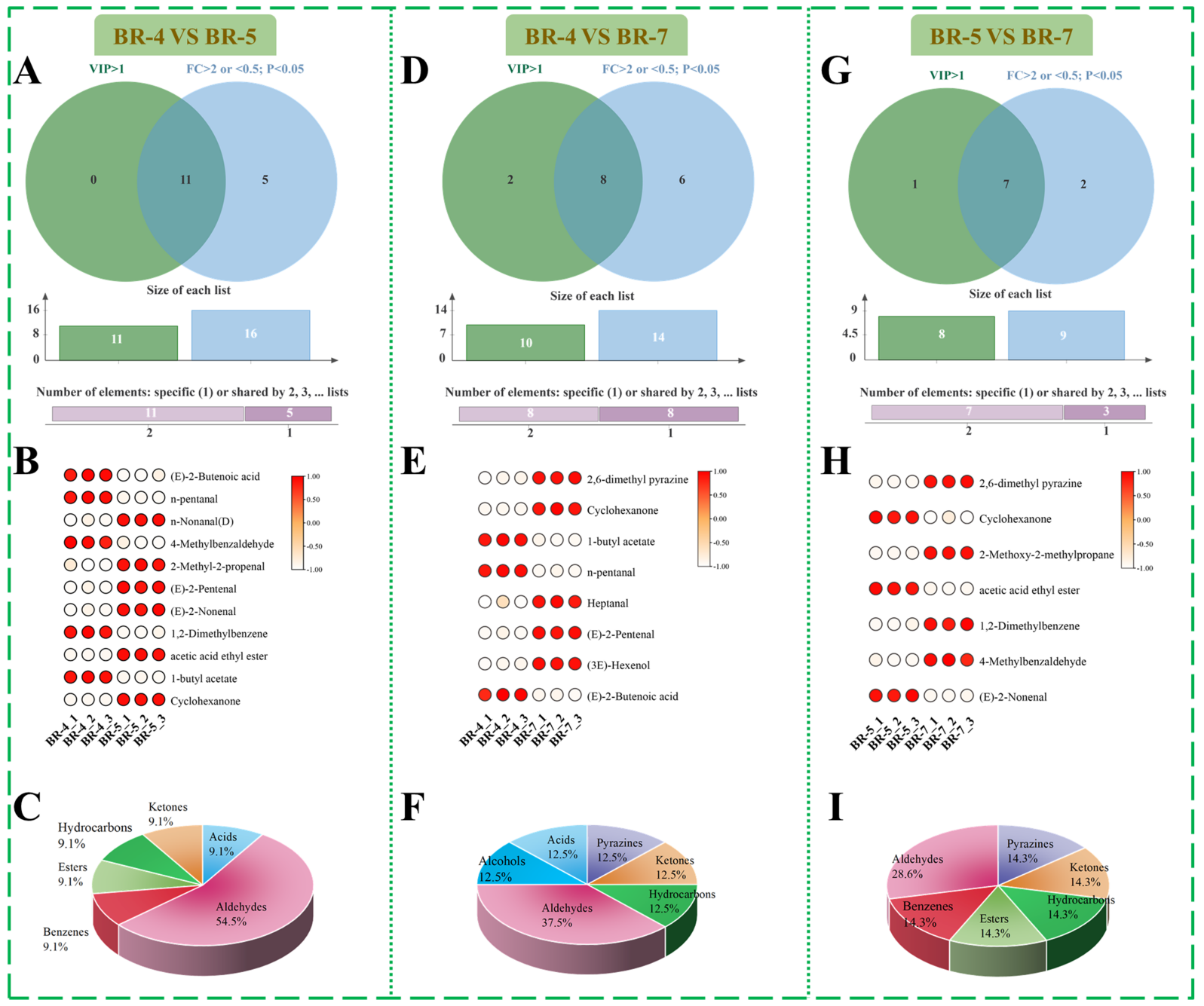
**

**Fig. S8.**

**
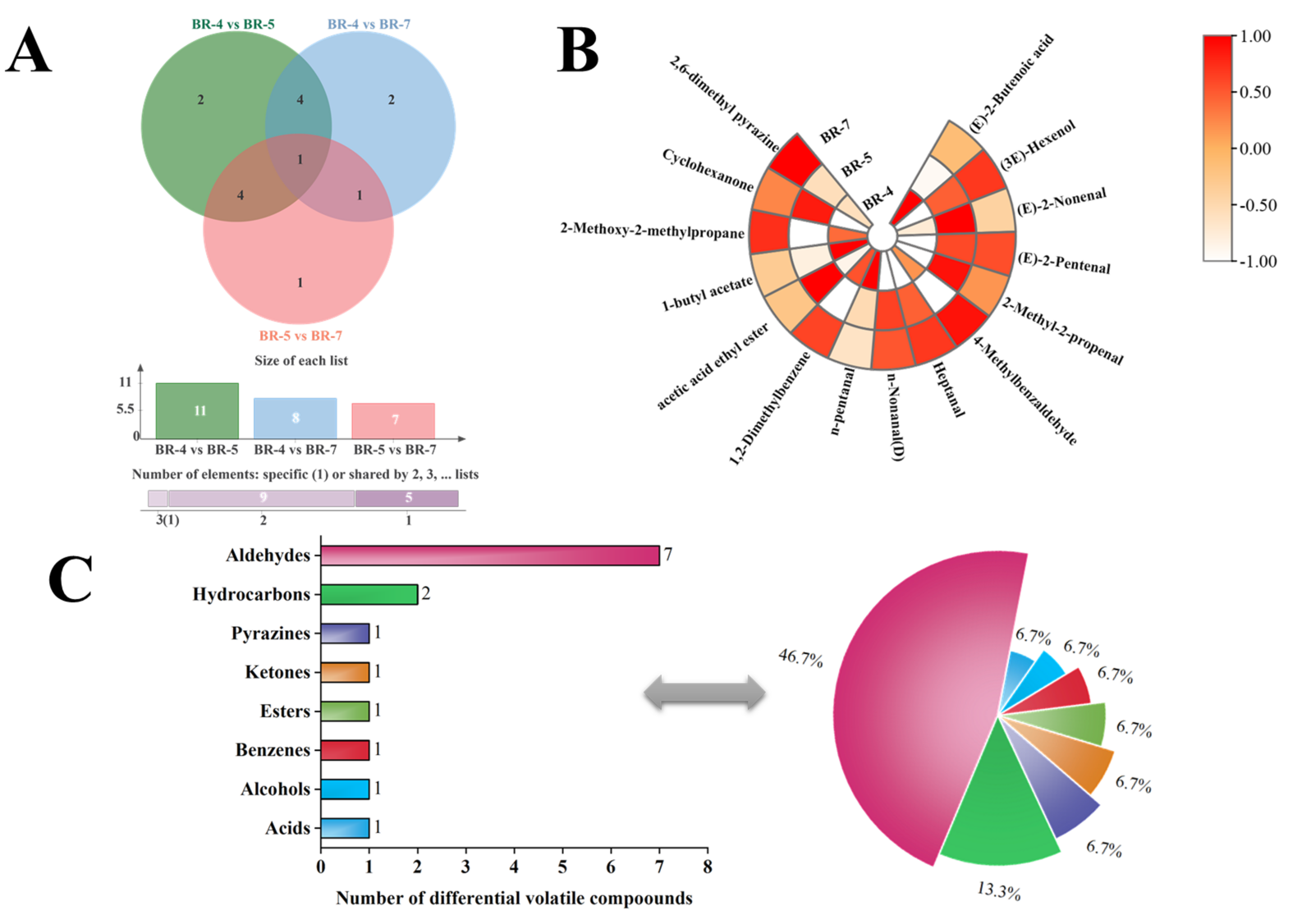
**

**Fig. S9.**

**Table S1. Volatile compounds characterized in rambutan seed oils using GC-MS**

| Compounds | Class | Retention Time (min) | Formula | RI |
| --- | --- | --- | --- | --- |
| 2-(Hydroxymethyl)-3-methyl-2-butenoic acid | Acids | 50.508 | C6H10O3 | 2078.936 |
| n-Propylmalonic acid | Acids | 49.197 | C6H10O4 | 2044.069 |
| Nonanoic acid | Acids | 50.398 | C9H18O2 | 2076.011 |
| D-Erythronic acid γ-lactone, diacetate | Acids | 25.692 | C8H10O6 | 1485.622 |
| 3-Methylbutanoic acid | Acids | 29.736 | C5H10O2 | 1583.317 |
| Methoxyacetic acid | Acids | 23.78 | C3H6O3 | 1438.06 |
| Acetic acid | Acids | 23.79 | C2H4O2 | 1438.308 |
| 2-Methylheptanoic acid | Acids | 21.621 | C8H16O2 | 1382.528 |
| Trimethylsilanol | Alcohols | 9.102 | C3H10OSi | 1033.405 |
| Phenol | Alcohols | 47 | C6H6O | 1984.438 |
| Ethanol | Alcohols | 6.768 | C2H6O | 931.942 |
| cis-1,4-Cyclohexanediol, O,O'-bis(trifluoroacetyl) | Alcohols | 4.98 | C10H10F6O4 | 732.353 |
| Benzeneethanol | Alcohols | 43.798 | C8H10O | 1893.477 |
| 4-Methylene-5-hexen-2-ol | Alcohols | 5.138 | C7H12O | 743.971 |
| 2-Octanol | Alcohols | 22.915 | C8H18O | 1416.542 |
| 2-Methyl-4-phenyl-2-butanol, TMS derivative | Alcohols | 28.66 | C14H24OSi | 1557.452 |
| 2-Butanol | Alcohols | 7.76 | C4H10O | 980.097 |
| 2,3,4-Trimethyl-1-pentanol | Alcohols | 50.562 | C8H18O | 2080.372 |
| 1-Hexanol | Alcohols | 20.37 | C6H14O | 1347.778 |
| Methyl glyoxal | Aldehydes | 15.121 | C3H4O2 | 1213.716 |
| α-Formylethylbenzene | Aldehydes | 19.263 | C9H10O | 1317.028 |
| 4-[(1-Phenyl-2-propen-1-yl)oxy]benzaldehyd | Aldehydes | 33.016 | C16H14O2 | 1646.096 |
| 2,4-Dimethylbenzaldehyde | Aldehydes | 39.696 | C9H10O | 1794.715 |
| Benzaldehyde | Aldehydes | 26.403 | C7H6O | 1503.197 |
| 2,5-Dihydroxybenzaldehyde, 2TMS derivative | Aldehydes | 26.835 | C13H22O3Si2 | 1513.582 |
| 2,4-Dihydroxybenzaldehyde, 2TMS derivative | Aldehydes | 7.237 | C13H22O3Si2 | 954.709 |
| 2-Aminopropanediamide | Amines | 4.913 | C3H7N3O2 | 727.426 |
| N-Allylbenzamide | Amines | 39.083 | C10H11NO | 1778.834 |
| 2-Propanamine | Amines | 7.646 | C3H9N | 974.563 |
| 2-Aminocyanoacetamide | Amines | 4.879 | C3H5N3O | 724.926 |
| 2-Amino-2-methyl-1-(pyrrolidin-1-yl)propan-1-one | Amines | 45.364 | C8H16N2O | 1937.291 |
| 1,5-Naphthyridin-4-amine | Amines | 33.009 | C8H7N3 | 1645.971 |
| 1,3-Butanediamine | Amines | 5.091 | C4H12N2 | 740.515 |
| p-Xylene | Benzenes | 11.6 | C8H10 | 1117.778 |
| o-Xylene | Benzenes | 13.404 | C8H10 | 1167.889 |
| o-Cymene | Benzenes | 19.967 | C10H14 | 1336.583 |
| Mesitylene | Benzenes | 17.358 | C9H12 | 1268.411 |
| Ethylbenzene | Benzenes | 13.639 | C8H10 | 1174.417 |
| 4-ethenyl-1,2-dimethyl-benzene | Benzenes | 24.736 | C10H12 | 1461.841 |
| 1-methyl-2-(1-methylethyl)benzene | Benzenes | 20.36 | C10H14 | 1347.5 |
| 1-Ethyl-4-methylbenzene | Benzenes | 14.923 | C9H12 | 1208.875 |
| 1-Ethyl-3,5-dimethylbenzene | Benzenes | 25.192 | C10H14 | 1473.184 |
| 1-Ethyl-2,4-dimethylbenzene | Benzenes | 23.197 | C10H14 | 1423.557 |
| 1,3-dimethyl- Benzene | Benzenes | 11.808 | C8H10 | 1123.556 |
| 1,3-Butadienylbenzene | Benzenes | 40.136 | C10H10 | 1805.659 |
| 1,2,3-Trimethylbenzene) | Benzenes | 17.117 | C9H12 | 1262.518 |
| 1,2,3,5-Tetramethylbenzene | Benzenes | 22.771 | C10H14 | 1412.96 |
| (1-Methylethyl)benzene | Benzenes | 17.331 | C9H12 | 1267.751 |
| Benzene | Benzenes | 6.795 | C6H6 | 933.252 |
| Tetradecanoic acid, 10,13-dimethyl-, methyl ester | Esters | 54.325 | C17H34O2 | 2155.915 |
| sec-Butyl acetate | Esters | 7.733 | C6H12O2 | 978.786 |
| Propanoic acid, ethenyl ester | Esters | 4.645 | C5H8O2 | 707.721 |
| Phthalic acid, 4-fluoro-2-nitrophenyl methyl ester | Esters | 56.226 | C15H10FNO6 | 2191.054 |
| Pentyl 3-methylbutanoate | Esters | 5.047 | C10H20O2 | 737.279 |
| Pentanoic acid, 1,1-dimethylpropyl ester | Esters | 5.611 | C10H20O2 | 778.75 |
| Oxalic acid, butyl cyclobutyl ester | Esters | 8.404 | C10H16O4 | 1008.387 |
| n-Propyl acetate | Esters | 9.974 | C5H10O2 | 1064.659 |
| N-Methyl-L-proline, pentyl ester | Esters | 6.275 | C11H21NO2 | 908.01 |
| Nitrous acid, ethyl ester | Esters | 23.787 | C2H5NO2 | 1438.234 |
| propyl 5-oxo-L-prolinate | Esters | 5.222 | C8H13NO3 | 750.147 |
| L-Alanine, ethyl ester | Esters | 10.265 | C5H11NO2 | 1075.09 |
| Dodecanoic acid, methyl ester | Esters | 39.764 | C13H26O2 | 1796.477 |
| Carbonic acid, ethyl-, methyl ester | Esters | 22.794 | C4H8O3 | 1413.532 |
| Butanoic acid, 2-cyano-3-methyl-, ethyl ester | Esters | 5 | C8H13NO2 | 733.824 |
| Butanethioic acid, 3-oxo-, S-(1,1-dimethylethyl) ester | Esters | 37.778 | C8H14O2S | 1745.026 |
| Borinic acid, diethyl-, methyl ester | Esters | 8.384 | C5H13BO | 1007.67 |
| Benzofenac methyl ester | Esters | 16.453 | C16H15ClO3 | 1246.284 |
| Acetic acid, butyl ester | Esters | 9.933 | C6H12O2 | 1063.19 |
| 3-Methyl-2-butenoic acid, cyclobutyl ester | Esters | 5.054 | C9H14O2 | 737.794 |
| 2-Methylbenzoic acid, 2,5-dichlorophenyl ester | Esters | 22.214 | C14H10Cl2O2 | 1399 |
| 2-Butenoic acid, methyl ester | Esters | 6.221 | C5H8O2 | 905.388 |
| Dibenzofuran | Furans | 54.915 | C12H8O | 2166.821 |
| 5-Methyl-2-(2-methyl-2-tetrahydrofuryl)tetrahydrofuran | Furans | 24.082 | C10H18O2 | 1445.572 |
| 3-methyl-(3H)-isobenzofuran-1-one | Furans | 25.631 | C9H8O2 | 1484.104 |
| 2-Acetyl-2-methyltetrahydrofuran | Furans | 4.229 | C7H12O2 | 624.146 |
| 3,4-Diacetylfurazan | Furans | 10.755 | C6H6N2O3 | 1092.652 |
| 2-Ethyltetrahydrofuran | Furans | 50.492 | C6H12O | 2078.511 |
| 2-Butyl-3-(4-.beta.-diethylaminoethoxybenzoyl)benzofuran | Furans | 4.678 | C25H31NO3 | 710.147 |
| 4,7-Dimethylundecane | Hydrocarbons | 11.107 | C13H28 | 1104.083 |
| 4,6-Dimethylundecane | Hydrocarbons | 13.995 | C13H28 | 1184.306 |
| 3,8-Dimethylundecane | Hydrocarbons | 16.322 | C13H28 | 1243.081 |
| Undecane | Hydrocarbons | 10.5 | C11H24 | 1083.513 |
| Styrene | Hydrocarbons | 16.154 | C8H8 | 1238.973 |
| Dimethyldimethoxysilane | Hydrocarbons | 5.064 | C4H12O2Si | 738.529 |
| 2-chloro-2-nitropropane | Hydrocarbons | 6.747 | C3H6ClNO2 | 930.922 |
| 2,3-Dimethylpentane | Hydrocarbons | 26.094 | C7H16 | 1495.622 |
| 2,3,3-Trimethylpentane | Hydrocarbons | 10.339 | C8H18 | 1077.742 |
| 3-Ethyl Octane | Hydrocarbons | 9.528 | C10H22 | 1048.674 |
| 2,3,6-trimethyloctane | Hydrocarbons | 8.454 | C11H24 | 1010.179 |
| 3,7-Dimethylnonane | Hydrocarbons | 9.219 | C11H24 | 1037.599 |
| 2,6-Dimethylnonane | Hydrocarbons | 8.421 | C11H24 | 1008.996 |
| Naphth[1,2-b]oxirene, 1a,2,3,7b-tetrahydro- | Hydrocarbons | 27.989 | C10H10O | 1541.322 |
| N,N'-Bis(2-methyl-2-nitrosopentan-4-one) | Hydrocarbons | 26.108 | C12H22N2O4 | 1495.97 |
| Trifluoroethene | Hydrocarbons | 5.014 | C2HF3 | 734.853 |
| Ethane, 2-chloro-1-(difluoromethoxy)-1,1,2-trifluoro | Hydrocarbons | 4.836 | C3H4ClF3O | 721.765 |
| 2,8,8-Trimethyldecane | Hydrocarbons | 16.235 | C13H28 | 1240.954 |
| 2-Methylenebutanenitrile | Hydrocarbons | 5.396 | C5H7N | 762.941 |
| 1,2-Dimethylazetidine | Hydrocarbons | 5.081 | C5H11N | 739.779 |
| 5-Bromo-1-hexene | Hydrocarbons | 21.812 | C6H11Br | 1387.833 |
| 3,5-Dimethyldodecane | Hydrocarbons | 18.334 | C14H30 | 1292.274 |
| 2,4,4-Trimethyl-2-pentene | Hydrocarbons | 5.278 | C8H16 | 754.265 |
| 2,2-dimethyl-1,3-dihydroindene | Hydrocarbons | 31.202 | C11H14 | 1613.761 |
| 2,3-Dihydro-5-methyl-1H-indene | Hydrocarbons | 25.628 | C10H12 | 1484.03 |
| 1,1-Dimethyl-2,3-dihydro-1H-indene | Hydrocarbons | 29.887 | C11H14 | 1586.947 |
| 1,3-Hexadien-5-yne | Hydrocarbons | 6.761 | C6H6 | 931.602 |
| (3E,7E)-4,8,12-Trimethyltrideca-1,3,7,11-tetraene | Hydrocarbons | 39.874 | C16H26 | 1799.326 |
| Homo-isomelodienone | Ketones | 15.232 | C16H16O5 | 1216.43 |
| Cyclohexanone | Ketones | 17.808 | C6H10O | 1279.413 |
| 5-(Cyclohexylmethyl)-2-pyrrolidinone | Ketones | 5.134 | C11H19NO | 743.676 |
| 2-Butanone | Ketones | 15.172 | C4H8O | 1214.963 |
| 3-Isopropylsydnone | Ketones | 26.587 | C9H8N2O2 | 1507.62 |
| 2'-Methylpropiophenone | Ketones | 23.67 | C10H12O | 1435.323 |
| 2-Methyl-3-heptanone | Ketones | 13.019 | C8H16O | 1157.194 |
| 2-Imidazolidinone | Ketones | 4.038 | C3H6N2O | 565.926 |
| 1-(4-Methylphenyl)-1-pentanone | Ketones | 20.222 | C12H16O | 1343.667 |
| 1-Cyclohexen-3-ol, 3-(2-propenyl)- | Ketones | 50.529 | C9H14O | 2079.495 |
| 2-Methylnaphthalene | Naphthalenes | 41.118 | C11H10 | 1829.208633 |
| 1-Methylnaphthalene | Naphthalenes | 41.112 | C11H10 | 1829.065 |
| 6-methyl-1,2,3,4-tetrahydronaphthalene | Naphthalenes | 26.597 | C10H12 | 1507.861 |
| 2-methyl-1,2,3,4-tetrahydronaphthalene | Naphthalenes | 27.939 | C11H14 | 1540.12 |
| Naphthalene | Naphthalenes | 36.289 | C10H8 | 1706.451 |
| (1E,2E)-2-Cyano-N-hydroxy-2-(hydroxyimino)ethanimidoyl chloride | Others | 52.735 | C3H2ClN3O2 | 2126.525 |
| Phenoxyacetonitrile | Others | 25.558 | C8H7NO | 1482.289 |
| Methyl tetradecanoate | Others | 47.668 | C15H30O2 | 2003.404 |
| Ammonium carbamate | Others | 4.219 | CH6N2O2 | 621.707 |
| 4-Hydrazinobenzonitrile | Others | 23.18 | C7H7N3 | 1423.134 |
| 3,5,5-Trimethylpyrazoline | Others | 5.409 | C6H12N2 | 763.897 |
| 2-Isopropenyl-3-methylpyrazine | Pyrazines | 39.693 | C8H10N2 | 1794.637 |
| 2,6-Dimethyl-3,5-dioxo-2,3,4,5-tetrahydro-1,2,4-triazine | Pyrazines | 42.487 | C5H7N3O2 | 1862.038 |
| 4-(2-Piperidin-1-yl-ethyl)-pyridine | Pyridines | 18.002 | C11H17N3 | 1284.156 |
| 1-(4-methylpent-3-en-1-yl)pyrrolidine | Pyrroles | 4.625 | C10H19N | 706.25 |
| Methanesulfonyl chloride | Sulfur compounds | 5.031 | CH3ClO2S | 736.103 |
| 3-dibenzo[b,e]thiepin-11(6H)-ylidene-N,N-dimethylpropylamine S-oxide | Sulfur compounds | 4.752 | C19H21NOS | 715.588 |

**Table S2. The statistical parameters of PCA and OPLS-DA modes of oil samples from three rambutan seed (GC-MS)**

| Mode | Group | R2Xcum | R2Ycum | Q2cum |
| --- | --- | --- | --- | --- |
| PCA | BR-4 & BR-5 & BR-7 | 0.997 |  | 0.994 |
| OPLS-DA | BR-4 & BR-5 & BR-7 | 0.997 | 1 | 1 |
|  | BR-4 & BR-5 | 0.998 | 1 | 1 |
|  | BR-4 & BR-7 | 0.999 | 1 | 1 |
|  | BR-5 & BR-7 | 0.999 | 1 | 1 |

**Table S3. Differential volatile compounds identified by GC-MS in the group of BR-4 vs BR-5**

| Component Name | Class | Retention Time | Formula | CAS No. | RI | VIP | *P*. value | Fold Change | Type |
| --- | --- | --- | --- | --- | --- | --- | --- | --- | --- |
| Pyrrolidine, N-(4-methyl-3-pentenyl)- | Pyrroles | 4.625 | C10H19N |  | 706.25 | 5.48392 | 2.39E-06 | 5.1683 | Up |
| Propanoic acid, ethenyl ester | Esters | 4.645 | C5H8O2 | 105-38-4 | 707.721 | 2.39546 | 2.16E-06 | 5.1679 | Up |
| Pentyl 3-methylbutanoate | Esters | 5.047 | C10H20O2 | 25415-62-7 | 737.279 | 1.161 | 8.41E-06 | 0.18979 | Down |
| Nonanoic acid | Acids | 50.398 | C9H18O2 | 112-05-0 | 2076.011 | 1.26828 | 1.13E-05 | 5.2752 | Up |
| Naphthalene | Naphthalenes | 36.289 | C10H8 | 91-20-3 | 1706.451 | 1.59701 | 3.09E-05 | 0.24437 | Down |
| propyl 5-oxo-L-prolinate | Esters | 5.222 | C8H13NO3 | | 750.147 | 4.18299 | 5.9E-08 | 5.0813 | Up |
| Ethanol | Alcohols | 6.768 | C2H6O | 64-17-5 | 931.942 | 1.34284 | 1.45E-06 | 5.16 | Up |
| Benzene, 1-ethyl-2,4-dimethyl- | Benzenes | 23.197 | C10H14 | 874-41-9 | 1423.557 | 1.60856 | 1.45E-05 | 0.065101 | Down |
| Benzene, 1,3-dimethyl- | Benzenes | 11.808 | C8H10 | 108-38-3 | 1123.556 | 1.33976 | 6.28E-07 | 0.19636 | Down |
| Benzene, 1,2,3,5-tetramethyl- | Benzenes | 22.771 | C10H14 | 527-53-7 | 1412.96 | 1.14155 | 5.28E-05 | 0.3323 | Down |
| Benzene | Benzenes | 6.795 | C6H6 | 71-43-2 | 933.252 | 1.003 | 1.5E-06 | 5.165 | Up |
| Azetidine, 1,2-dimethyl- | Hydrocarbons | 5.081 | C5H11N | 51764-32-0 | 739.779 | 1.54478 | 1.32E-07 | 5.0998 | Up |
| 3-Heptanone, 2-methyl- | Ketones | 13.019 | C8H16O | 13019-20-0 | 1157.194 | 1.46044 | 6.04E-07 | 0.19651 | Down |
| 2-Octanol | Alcohols | 22.915 | C8H18O | 123-96-6 | 1416.542 | 1.69596 | 1.69E-05 | 0.18708 | Down |
| 2-Imidazolidinone | Ketones | 4.038 | C3H6N2O | 120-93-4 | 565.926 | 1.39954 | 7.14E-06 | 5.2363 | Up |
| 2-Butyl-3-(4-.beta.-diethylaminoethoxybenzoyl)benzofuran | Furans | 4.678 | C25H31NO3 | 23551-25-9 | 710.147 | 1.28373 | 2.44E-06 | 5.2065 | Up |

**Table S4. Differential volatile compounds identified by GC-MS in the group of BR-4 vs BR-7**

| Component Name | Class | Retention Time | Formula (mol ion) | CAS No. | RI | VIP | *P*. value | Fold Change | Type |
| --- | --- | --- | --- | --- | --- | --- | --- | --- | --- |
| sec-Butyl acetate | Esters | 7.733 | C6H12O2 | 105-46-4 | 978.786 | 2.48723 | 2.31E-05 | 0.18889 | Down |
| Pyrrolidine, N-(4-methyl-3-pentenyl)- | Pyrroles | 4.625 | C10H19N |  | 706.25 | 3.61325 | 2.39E-06 | 5.1683 | Up |
| Propanoic acid, ethenyl ester | Esters | 4.645 | C5H8O2 | 105-38-4 | 707.721 | 1.57832 | 2.16E-06 | 5.1679 | Up |
| Pentyl 3-methylbutanoate | Esters | 5.047 | C10H20O2 | 25415-62-7 | 737.279 | 2.13501 | 9.23E-06 | 0.18881 | Down |
| Oxalic acid, butyl cyclobutyl ester | Esters | 8.404 | C10H16O4 | | 1008.387 | 1.76647 | 1.52E-05 | 0.02161 | Down |
| propyl 5-oxo-L-prolinate | Esters | 5.222 | C8H13NO3 | | 750.147 | 2.75625 | 5.9E-08 | 5.0813 | Up |
| Butanoic acid, 2-cyano-3-methyl-, ethyl ester | Esters | 5 | C8H13NO2 | 3213-49-8 | 733.824 | 1.12248 | 8.47E-06 | 0.18973 | Down |
| Azetidine, 1,2-dimethyl- | Hydrocarbons | 5.081 | C5H11N | 51764-32-0 | 739.779 | 1.01788 | 1.32E-07 | 5.0998 | Up |
| 2-Pyrrolidinone, 5-(cyclohexylmethyl)- | Ketones | 5.134 | C11H19NO | 14293-08-4 | 743.676 | 5.61608 | 8.44E-06 | 0.18974 | Down |
| 2-Aminocyanoacetamide | Amines | 4.879 | C3H5N3O | 6719-21-7 | 724.926 | 3.2148 | 2.22E-05 | 0.18706 | Down |

**Table S5. Differential volatile compounds identified by GC-MS in the group of BR-5 vs BR-7**

| Component Name | Class | Retention Time | Formula (mol ion) | CAS No. | RI | VIP | *P*. value | Fold Change | Type |
| --- | --- | --- | --- | --- | --- | --- | --- | --- | --- |
| sec-Butyl acetate | Esters | 7.733 | C6H12O2 | 105-46-4 | 978.786 | 2.67985 | 5.59E-05 | 0.3463 | Down |
| Pentyl 3-methylbutanoate | Esters | 5.047 | C10H20O2 | 25415-62-7 | 737.279 | 2.39219 | 7.16E-06 | 0.12867 | Down |
| Oxalic acid, butyl cyclobutyl ester | Esters | 8.404 | C10H16O4 | | 1008.387 | 2.14387 | 3.24E-05 | 0.19087 | Down |
| Naphthalene | Naphthalenes | 36.289 | C10H8 | 91-20-3 | 1706.451 | 1.18326 | 5.99E-05 | 2.969 | Up |
| Butanoic acid, 2-cyano-3-methyl-, ethyl ester | Esters | 5 | C8H13NO2 | 3213-49-8 | 733.824 | 1.34735 | 8.47E-06 | 0.18973 | Down |
| Benzene, 1-ethyl-2,4-dimethyl- | Benzenes | 23.197 | C10H14 | 874-41-9 | 1423.557 | 1.23974 | 1.78E-05 | 9.0166 | Up |
| Benzene, 1,3-dimethyl- | Benzenes | 11.808 | C8H10 | 108-38-3 | 1123.556 | 1.05948 | 6.28E-07 | 5.0926 | Up |
| Benzene | Benzenes | 6.795 | C6H6 | 71-43-2 | 933.252 | 1.0552 | 1.88E-05 | 0.19319 | Down |
| 3-Heptanone, 2-methyl- | Ketones | 13.019 | C8H16O | 13019-20-0 | 1157.194 | 1.15524 | 6.04E-07 | 5.0889 | Up |
| 2-Pyrrolidinone, 5-(cyclohexylmethyl)- | Ketones | 5.134 | C11H19NO | 14293-08-4 | 743.676 | 6.74116 | 8.44E-06 | 0.18974 | Down |
| 2-Octanol | Alcohols | 22.915 | C8H18O | 123-96-6 | 1416.542 | 1.34173 | 1.69E-05 | 5.3454 | Up |
| 2-Aminocyanoacetamide | Amines | 4.879 | C3H5N3O | 6719-21-7 | 724.926 | 3.85886 | 2.22E-05 | 0.18706 | Down |

**Table S6. Differential volatile compounds identified by GC-MS in rambutan seed oils**

| Component Name | Index | Class | Retention Time | Formula (mol ion) | CAS No. |
| --- | --- | --- | --- | --- | --- |
| sec-Butyl acetate | DVOCs-1 | Esters | 7.733 | C6H12O2 | 105-46-4 |
| Pyrrolidine, N-(4-methyl-3-pentenyl)- | DVOCs-2 | Pyrroles | 4.625 | C10H19N |  |
| Propanoic acid, ethenyl ester | DVOCs-3 | Esters | 4.645 | C5H8O2 | 105-38-4 |
| Pentyl 3-methylbutanoate | DVOCs-4 | Esters | 5.047 | C10H20O2 | 25415-62-7 |
| Oxalic acid, butyl cyclobutyl ester | DVOCs-5 | Esters | 8.404 | C10H16O4 |  |
| Nonanoic acid | DVOCs-6 | Acids | 50.398 | C9H18O2 | 112-05-0 |
| Naphthalene | DVOCs-7 | Naphthalenes | 36.289 | C10H8 | 91-20-3 |
| propyl 5-oxo-L-prolinate | DVOCs-8 | Esters | 5.222 | C8H13NO3 | |
| Ethanol | DVOCs-9 | Alcohols | 6.768 | C2H6O | 64-17-5 |
| Butanoic acid, 2-cyano-3-methyl-, ethyl ester | DVOCs-10 | Esters | 5.000 | C8H13NO2 | 3213-49-8 |
| Benzene, 1-ethyl-2,4-dimethyl- | DVOCs-11 | Benzenes | 23.197 | C10H14 | 874-41-9 |
| Benzene, 1,3-dimethyl- | DVOCs-12 | Benzenes | 11.808 | C8H10 | 108-38-3 |
| Benzene, 1,2,3,5-tetramethyl- | DVOCs-13 | Benzenes | 22.771 | C10H14 | 527-53-7 |
| Benzene | DVOCs-14 | Benzenes | 6.795 | C6H6 | 71-43-2 |
| Azetidine, 1,2-dimethyl- | DVOCs-15 | Hydrocarbons | 5.081 | C5H11N | 51764-32-0 |
| 3-Heptanone, 2-methyl- | DVOCs-16 | Ketones | 13.019 | C8H16O | 13019-20-0 |
| 2-Pyrrolidinone, 5-(cyclohexylmethyl)- | DVOCs-17 | Ketones | 5.134 | C11H19NO | 14293-08-4 |
| 2-Octanol | DVOCs-18 | Alcohols | 22.915 | C8H18O | 123-96-6 |
| 2-Imidazolidinone | DVOCs-19 | Ketones | 4.038 | C3H6N2O | 120-93-4 |
| 2-Butyl-3-(4-.beta.-diethylaminoethoxybenzoyl)benzofuran | DVOCs-20 | Furans | 4.678 | C25H31NO3 | 23551-25-9 |
| 2-Aminocyanoacetamide | DVOCs-21 | Amines | 4.879 | C3H5N3O | 6719-21-7 |

**Table S7. Volatile compounds characterized in rambutan seed oils using GC-IMS**

| Compounds | Class | CAS | Formula | RI | Rt [sec] |
| --- | --- | --- | --- | --- | --- |
| (E)-2-Butenoic acid | Acids | C107937 | C4H6O2 | 871.9 | 660.282 |
| Butanoic acid | Acids | C107926 | C4H8O2 | 842 | 763.852 |
| Propanoic acid | Acids | C79094 | C3H6O2 | 674.4 | 246.223 |
| (3E)-Hexenol | Alcohols | C928972 | C6H12O | 813.3 | 545.829 |
| 1-butanol 2-methyl | Alcohols | C137326 | C5H12O | 711.2 | 345.209 |
| 1-Hexanol | Alcohols | C111273 | C6H14O | 871.4 | 659.182 |
| 1-Hexanol, 2-ethyl- | Alcohols | C104767 | C8H18O | 1008.4 | 1128.714 |
| 1-Pentanol | Alcohols | C71410 | C5H12O | 760.1 | 599.093 |
| 2-Butanol | Alcohols | C78922 | C4H10O | 614.4 | 369.776 |
| 2-Methyl-3-furanthiol | Alcohols | C28588741 | C5H6OS | 873.5 | 663.585 |
| 3-Methyl-3-buten-1-ol | Alcohols | C763326 | C5H10O | 712.1 | 347.901 |
| (E)-2-Nonenal | Aldehydes | C18829566 | C9H16O | 1146.3 | 1231.941 |
| (E)-2-Pentenal | Aldehydes | C1576870 | C5H8O | 719.7 | 691.668 |
| 1-hexanal(D) | Aldehydes | C66251 | C6H12O | 794.9 | 444.484 |
| 1-hexanal(M) | Aldehydes | C66251 | C6H12O | 794.9 | 514.352 |
| 2-Methyl-2-propenal | Aldehydes | C78853 | C4H6O | 556.3 | 382.742 |
| 2-Methylpentanal | Aldehydes | C123159 | C6H12O | 756.7 | 441.401 |
| 4-Methylbenzaldehyde | Aldehydes | C104870 | C8H8O | 1115.2 | 1157.195 |
| Heptanal | Aldehydes | C111717 | C7H14O | 903.3 | 724.614 |
| n-Nonanal(D) | Aldehydes | C124196 | C9H18O | 1102.8 | 723.816 |
| n-Nonanal(M) | Aldehydes | C124196 | C9H18O | 1102.7 | 1128.491 |
| n-Octanal | Aldehydes | C124130 | C8H16O | 1008.4 | 933.511 |
| n-pentanal | Aldehydes | C110623 | C5H10O | 696.1 | 316.97 |
| 1,2-Dimethylbenzene | Benzenes | C95476 | C8H10 | 924.7 | 933.528 |
| acetic acid ethyl ester | Esters | C141786 | C4H8O2 | 571 | 248.809 |
| ethyl acrylate | Esters | C140885 | C5H8O2 | 694.2 | 236.709 |
| Propyl butanoate | Esters | C105668 | C7H14O2 | 902.8 | 1231.941 |
| 1-butyl acetate | Hydrocarbons | C123864 | C6H12O2 | 813.3 | 514.352 |
| 2-Methoxy-2-methylpropane | Hydrocarbons | C1634044 | C5H12O | 574.9 | 276.616 |
| 1-Hydroxy-2-propanone | Ketones | C116096 | C3H6O2 | 665.2 | 324.83 |
| 2,5-dimethyl-4-hydroxy 3(2H)- furanone (Furaneol) | Ketones | C3658773 | C6H8O3 | 1111.5 | 1148.552 |
| 2-Heptanone | Ketones | C110430 | C7H14O | 886.2 | 450.633 |
| 4-methyl-2-pentanone | Ketones | C108101 | C6H12O | 755 | 371.191 |
| Cyclohexanone | Ketones | C108941 | C6H10O | 895.3 | 710.502 |
| 2,6-dimethyl pyrazine | Pyrazines | C108509 | C6H8N2 | 895.3 | 710.557 |

**Table S8. The statistical parameters of PCA and OPLS-DA modes of oil samples from three rambutan seed (GC-IMS)**

| Mode | Group | R^2^X_cum_ | R^2^Y_cum_ | Q^2^_cum_ |
| --- | --- | --- | --- | --- |
| PCA | BR-4 & BR-5 & BR-7 | 0.837 |  | 0.705 |
| OPLS-DA | BR-4 & BR-5 & BR-7 | 0.837 | 0.996 | 0.989 |
|  | BR-4 & BR-5 | 0.925 | 1 | 0.999 |
|  | BR-4 & BR-7 | 0.981 | 1 | 0.999 |
|  | BR-5 & BR-7 | 0.883 | 0.999 | 0.996 |

**Table S9. Differential volatile compounds identified using GC-IMS in the group of BR-4 vs BR-5**

| Compound | Class | Formula | VIP | *P.* value | Fold Change | Type |
| --- | --- | --- | --- | --- | --- | --- |
| (E)-2-Butenoic acid | Acids | C4H6O2 | 1.60711 | 5.31E-05 | 8.4789 | Up |
| n-pentanal | Aldehydes | C5H10O | 2.00242 | 5.21E-06 | 7.9589 | Up |
| n-Nonanal(D) | Aldehydes | C9H18O | 1.00417 | 1.84E-07 | 0.056997 | Down |
| 4-Methylbenzaldehyde | Aldehydes | C8H8O | 1.01731 | 0.026369 | 2.701 | Up |
| 2-Methyl-2-propenal | Aldehydes | C4H6O | 1.14271 | 5.21E-06 | 0.34763 | Down |
| (E)-2-Pentenal | Aldehydes | C5H8O | 1.09616 | 2.55E-06 | 0.13856 | Down |
| (E)-2-Nonenal | Aldehydes | C9H16O | 1.69696 | 1.64E-05 | 0.069534 | Down |
| 1,2-Dimethylbenzene | Benzenes | C8H10 | 1.2392 | 6.42E-06 | 10.119 | Up |
| acetic acid ethyl ester | Esters | C4H8O2 | 1.73251 | 3.26E-07 | 0.10016 | Down |
| 1-butyl acetate | Hydrocarbons | C6H12O2 | 2.05285 | 3.75E-06 | 11.171 | Up |
| Cyclohexanone | Ketones | C6H10O | 2.27939 | 1.31E-07 | 0.086699 | Down |

**Table S10. Differential volatile compounds identified using GC-IMS in the group of BR-4 vs BR-7**

| Compound | Class | Formula | VIP | *P.* value | Fold Change | Type |
| --- | --- | --- | --- | --- | --- | --- |
| 2,6-dimethyl pyrazine | Pyrazines | C6H8N2 | 2.29594 | 2.22E-07 | 0.16039 | Down |
| Cyclohexanone | Ketones | C6H10O | 1.59437 | 8.52E-06 | 0.18215 | Down |
| 1-butyl acetate | Hydrocarbons | C6H12O2 | 2.10714 | 5.39E-06 | 6.0694 | Up |
| n-pentanal | Aldehydes | C5H10O | 2.16695 | 4.84E-06 | 9.2192 | Up |
| Heptanal | Aldehydes | C7H14O | 1.07733 | 0.000136 | 0.49505 | Down |
| (E)-2-Pentenal | Aldehydes | C5H8O | 1.15146 | 1.05E-06 | 0.14344 | Down |
| (3E)-Hexenol | Alcohols | C6H12O | 1.05844 | 6.58E-08 | 0.079174 | Down |
| (E)-2-Butenoic acid | Acids | C4H6O2 | 1.55515 | 0.000115 | 3.58 | Up |

**Table S11. Differential volatile compounds identified using GC-IMS in the group of BR-5 vs BR-7**

| Compound | Class | Formula | VIP | *P*. value | Fold Change | Type |
| --- | --- | --- | --- | --- | --- | --- |
| 2,6-dimethyl pyrazine | Pyrazines | C6H8N2 | 2.56999 | 1.63E-07 | 0.16205 | Down |
| Cyclohexanone | Ketones | C6H10O | 2.07252 | 7.32E-06 | 2.101 | Up |
| 2-Methoxy-2-methylpropane | Hydrocarbons | C5H12O | 1.06593 | 1.68E-09 | 0.17714 | Down |
| acetic acid ethyl ester | Esters | C4H8O2 | 1.94778 | 5.79E-07 | 4.7853 | Up |
| 1,2-Dimethylbenzene | Benzenes | C8H10 | 1.5825 | 5.61E-05 | 0.088432 | Down |
| 4-Methylbenzaldehyde | Aldehydes | C8H8O | 1.85772 | 0.002121 | 0.20824 | Down |
| (E)-2-Nonenal | Aldehydes | C9H16O | 1.99168 | 1.96E-05 | 8.9457 | Up |

**Table S12. Differential volatile compounds identified using GC-IMS in rambutan seed oils**

| Compound | Class | Formula | RI | Rt [sec] |
| --- | --- | --- | --- | --- |
| (E)-2-Butenoic acid | Acids | C4H6O2 | 871.9 | 660.282 |
| (3E)-Hexenol | Alcohols | C6H12O | 813.3 | 545.829 |
| (E)-2-Nonenal | Aldehydes | C9H16O | 1146.3 | 1231.941 |
| (E)-2-Pentenal | Aldehydes | C5H8O | 719.7 | 691.668 |
| 2-Methyl-2-propenal | Aldehydes | C4H6O | 556.3 | 382.742 |
| 4-Methylbenzaldehyde | Aldehydes | C8H8O | 1115.2 | 1157.195 |
| Heptanal | Aldehydes | C7H14O | 903.3 | 724.614 |
| n-Nonanal(D) | Aldehydes | C9H18O | 1102.8 | 723.816 |
| n-pentanal | Aldehydes | C5H10O | 696.1 | 316.97 |
| 1,2-Dimethylbenzene | Benzenes | C8H10 | 924.7 | 933.528 |
| acetic acid ethyl ester | Esters | C4H8O2 | 571 | 248.809 |
| 1-butyl acetate | Hydrocarbons | C6H12O2 | 813.3 | 514.352 |
| 2-Methoxy-2-methylpropane | Hydrocarbons | C5H12O | 574.9 | 276.616 |
| Cyclohexanone | Ketones | C6H10O | 895.3 | 710.502 |
| 2,6-dimethyl pyrazine | Pyrazines | C6H8N2 | 895.3 | 710.557 |
